# Quercetin attenuates neurotoxicity induced by iron oxide nanoparticles

**DOI:** 10.1186/s12951-021-01059-0

**Published:** 2021-10-18

**Authors:** Akram Bardestani, Shiva Ebrahimpour, Ali Esmaeili, Abolghasem Esmaeili

**Affiliations:** 1 Department of Cell and Molecular Biology & Microbiology, Faculty of Biological Science and Technology, University of Isfahan, P.O. Box: 8174673441, Isfahan, Iran; 2 School of Dentistry, Isfahan University of Medical Sciences, Isfahan, Iran

**Keywords:** Quercetin, Iron oxide nanoparticles, Neurotoxicity, Iron overload, Neurodegenerative diseases

## Abstract

Iron oxide nanoparticles (IONPs) have been proposed as targeted carriers to deliver therapeutic molecules in the central nervous system (CNS). However, IONPs may damage neural tissue via free iron accumulation, protein aggregation, and oxidative stress. Neuroprotective effects of quercetin (QC) have been proven due to its antioxidant and anti-inflammatory properties. However, poor solubility and low bioavailability of QC have also led researchers to make various QC-involved nanoparticles to overcome these limitations. We wondered how high doses or prolonged treatment with quercetin conjugated superparamagnetic iron oxide nanoparticles (QCSPIONs) could improve cognitive dysfunction and promote neurogenesis without any toxicity. It can be explained that the QC inhibits protein aggregation and acts against iron overload via iron-chelating activity, iron homeostasis genes regulation, radical scavenging, and attenuation of Fenton/Haber–Weiss reaction. In this review, first, we present brain iron homeostasis, molecular mechanisms of iron overload that induced neurotoxicity, and the role of iron in dementia-associated diseases. Then by providing evidence of IONPs neurotoxicity, we discuss how QC neutralizes IONPs neurotoxicity, and finally, we make a brief comparison between QC and conventional iron chelators. In this review, we highlight that QC as supplementation and especially in conjugated form reduces iron oxide nanoparticles neurotoxicity in clinical application.

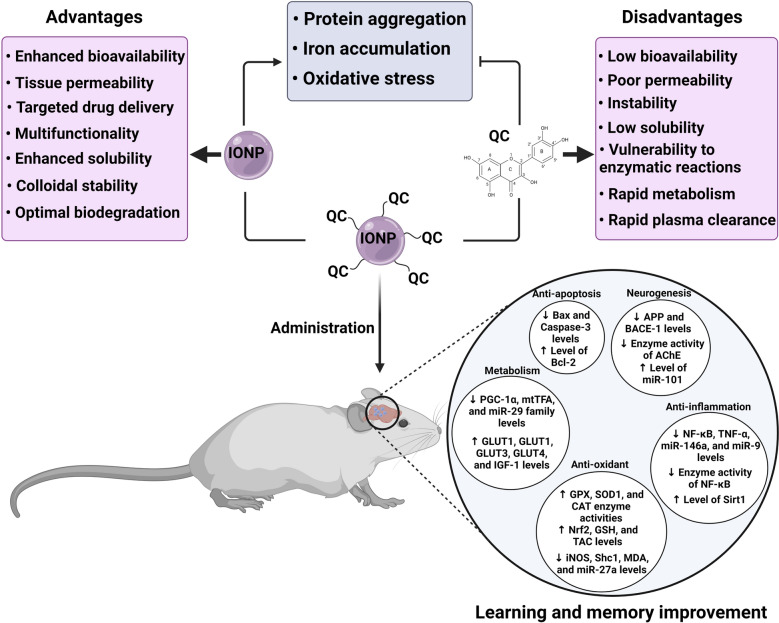

## Background

The term nanomaterial refers to material in the nanoscale (1–100 nm) with one, two, or three external dimensions, whereas the term nanoparticle (NP) refers to materials with all three external dimensions in the nanoscale [1]. The most important properties of NPs are the high surface to mass ratio, catalytic activity, electrical and thermal conductivity, high solubility, and mobility in the body tissues [2]. There are two general categories of biomedicine NPs. (I) Organic NPs that are mainly composed of organic molecules. Liposomes, emulsions, dendrimers, and other polymers form a large group of organic NPs. (II) Inorganic NPs that consist of a metal core such as iron, nickel, cobalt, gold, silica, and iron oxides with electrical, magnetic, optical, and fluorescent properties [3]. Iron oxide nanoparticles (IONPs) are a class of magnetic nanoparticles (MNPs) that have gained prominence in technological advancements [4, 5]. IONPs are usually made of maghemite (γ-Fe_2_O_3_) or magnetite (Fe_3_O_4_) core and a protective coating such as chitosan, dextran, polyethylene glycol (PEG), and polyvinyl alcohol (PVA) [6–8]. IONPs have unique properties that make them suitable biomaterials for medical applications. For instance, their Ferro-or ferromagnetic behavior enables drug trafficking and drug guidance to the target tissue. They can be localized in specific tissue under an external magnetic field so that they are called magnetic targeted carriers (MTC) [9]. Moreover, the application of IONPs in magnetic resonance imaging (MRI) is a powerful tool for creating high contrast medical images and enhances the potential of disease diagnosis [6, 10]. Besides, IONPs can make cancer cells more susceptible to radiation and chemotherapy by rising tumor temperature (hyperthermia). Furthermore, the ability of IONPs to cross the blood–brain barrier (BBB) is a privileged property for transporting drugs to the brain in neurological disorders [6–8]. At present, there are numerous FDA-approved SPION compounds (including, ferumoxide (Feridex I.V.), ferumoxsil (Lumirem), and ferumoxytol (Feraheme)), for use in the clinic and others undergoing clinical trials, as well as numbers of applicable IONPs, are ongoing. Despite the advantages mentioned above, in vitro, and in vivo studies provided evidence concerning the possible neurotoxicity of IONPs due to free iron accumulation, ROS production, and protein aggregation [11–14]. However, modifying the physicochemical properties of NPs such as concentration, size, and surface coating can optimize their function and cytotoxicity properties [14]. Besides, the simultaneous use of natural antioxidants such as quercetin (QC) supplementation can be a useful path to remove brain oxidative damages due to IONPs [15]. QC (3,3′,4′,5,7-pentahydroxyflavone) belongs to the flavonoid class and flavonol subclass with the chemical formula of C15H10O7 [16]. QC is a major component in many fruits, seeds, vegetables, and nuts. The beneficial effects of QC have been investigated in many disorders such as cancer and neurodegenerative diseases [17, 18]. QC can postpone or prevent neurodegenerative disease through multiple molecular pathways [16, 19]. QC modulates oxidative stress status via binding to the reactive oxygen and/or nitrogen species (ROS/RNS) and via its effect on the expression and activity of enzymatic/non-enzymatic antioxidants [15, 20]. QC also prevents the Fenton reaction by creating stable iron-QC complexes, thereby indirectly removing ROS/RNS [18]. Moreover, in iron overload conditions QC, can regulate iron homeostasis [21]. QC cannot cross the BBB well due to its low solubility, instability, and low bioavailability [20]. The use of IONPs is an effective solution to overcome these limitations [22]. Therefore, combining QC with IONPs is a mutually beneficial solution to neutralize iron toxicity and increase QC bioavailability. The beneficial effects of QC against IONPs-induced neurotoxicity are poorly defined. In this review, we provide evidence that QC can act against iron overload-induced toxicity. This iron overload can be caused by IONPs metabolism or other sources. However, QC likely has equal activities for neutralizing excess iron arisen from various sources.

## Iron metabolism and homeostasis

**Fig. 1:**
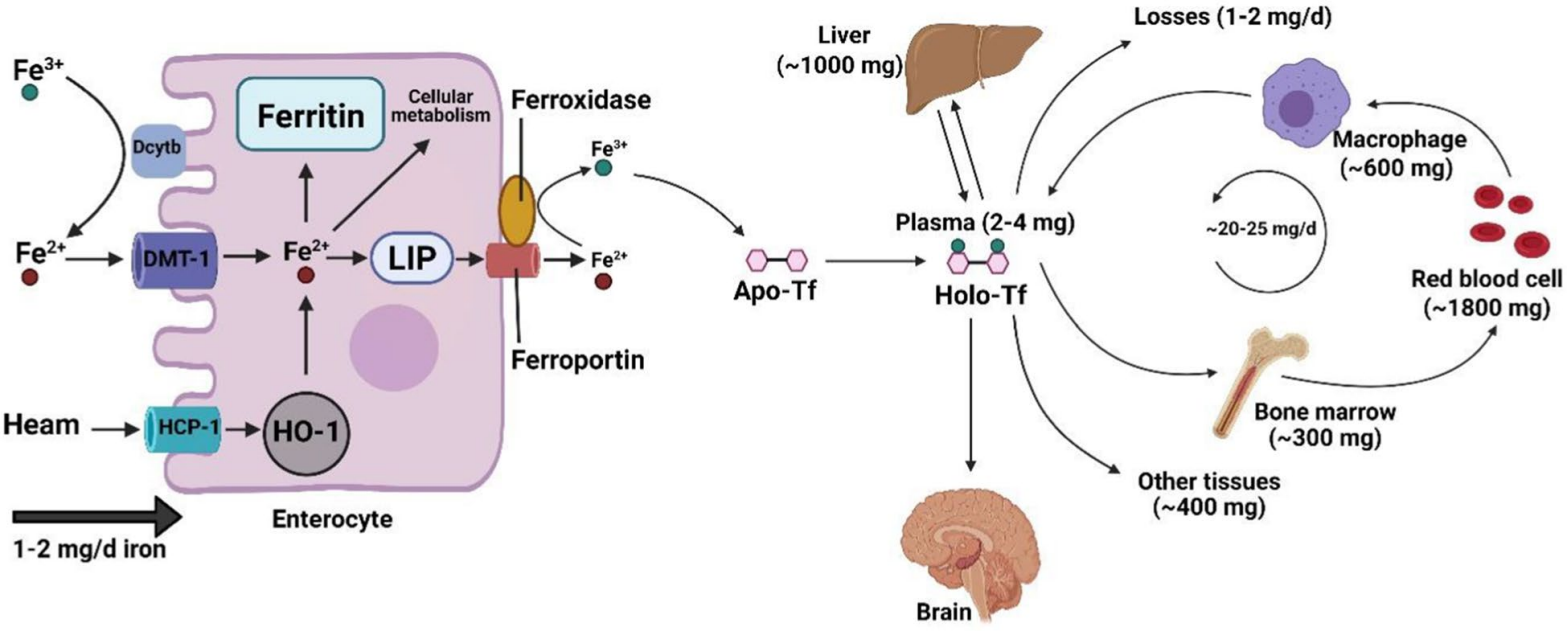
Iron metabolism and homeostasis: DMT1 and HCP1 transfer Fe^2+^ and heme from the gut lumen into the enterocytes. Fe^2+^ arising from non-heme and heme iron enters the LIP. Fe^2+^ can release from the cell by iron exporter ferroportin and the surplus of that transfer to a cytosolic iron storage protein called ferritin. The iron released from the enterocytes is re-oxidized to Fe^3+^ and transport by plasma iron-free transferrin so-called Apo-Tf. Apo-Tf can bind to ferric ions and form Holo-Tf and deliver iron to the target tissues such as bone marrow, liver, and brain. DMT1, Divalent metal transporter 1; HCP1, heme carrier protein 1; LIP, labile iron pool; Apo-Tf, apo-transferrin; Holo-Tf, holo-transferrin; HO-1, heme oxygenase-1; Dcytb, duodenal cytochrome B. This Figure was created by BioRender (https://biorender.com/)

Iron in our body is an essential mineral for many fundamental processes such as oxygen transport and mitochondrial function. Iron, also as a co-factor, participates in enzymatic reactions including, DNA replication, RNA transcription, protein translation, and myelin synthesis [23, 24]. The human body contains about 3–4 g of iron which may be lost up to 0.1% daily under physiological and pathological conditions that are usually compensated with daily dietary consumption [25]. Both iron deficiency and iron overload can affect the development and function of the brain from fetal to adulthood [26–28]. There are two forms of iron in daily diet: heme iron with absorbable ferrous ion (Fe^2+^) that exists in red meat and seafood, and non-heme iron with ferric ion (Fe^3+^) that exists in plant-based foods [29, 30]. Iron absorption can be controlled through body iron levels and multiple iron regulatory agents [27]. Duodenal cytochrome B (Dcytb) is an ascorbate-dependant plasma transmembrane ferrireductase that shifts Fe^3+^ to Fe^2+^ on the apical membranes of intestinal absorptive cells, enterocytes [31]. Iron enters the cell through metal transporters [32]. Divalent metal transporter 1 (DMT1) and heme carrier protein 1 (HCP1) are the main non-heme and heme iron transporters, respectively. They can transfer Fe^2+^ and heme from the gut lumen into the enterocytes [29, 30, 32]. HCP1 is preferably the high-affinity obligatory folate transporter [33]. In the next step, Fe^2+^ arising from non-heme and heme iron degraded by heme oxygenase-1 (HO-1) enter the labile iron pool (LIP), a transient intracellular iron pool [23]. The majority of this Fe^2+^ is released from the cell by iron exporter ferroportin in the basolateral membrane of enterocytes [34]. Its surplus is transferred to a cytosolic iron-storage protein called ferritin. Intestinal ferritin is an effective factor in iron absorption due to the ferroxidase activity of its H subunit that re-oxidizes Fe^2+^ to Fe^3+^ [23, 35, 36]. On other hand, the iron released from the enterocytes is re-oxidized to Fe^3+^ by ferroxidases (i.e., membrane-bound multicopper hephaestin and soluble and/membrane-bound multicopper ceruloplasmin), which are involved in the iron export by ferroportin [37, 38]. Iron oxidation is essential for iron transfer by plasma iron-free transferrin, so-called apo-transferrin (Apo-Tf). Trapping and retaining Fe^3+^ by iron-storage proteins such as ferritin and transferrin suppresses Fe^3+^ reactivity and free radical generation [39]. Apo-Tf binds to two ferric ions at normal alkaline pH (7.4) of the plasma to form holo-transferrin (Holo-Tf). This iron-loaded glycoprotein as a plasma iron pool delivers iron to the target tissues such as bone marrow, liver, and brain [25, 40, 41] (Fig. 1). Hepatocytes and macrophages are responsible for iron storage and iron recycling, respectively [42]. Under physiological conditions, approximately the whole of the extracellular iron enters the target cell in the form of transferrin-bound. However, transferrin saturation due to iron overload prevents iron binding to transferrin and leads to non-transferrin bound iron (NTBI) uptake [43]. Holo-Tf binds to transferrin receptor (TfR) on the surface of most cells [44]. The Holo-Tf-TfR complex is internalized to the cell via clathrin-coated vesicles along with adaptor protein 2 (AP2) in the endocytosis cycle termed clathrin-mediated endocytosis (CME) [45, 46]. The endocytic vesicles lose their clathrin coating and subsequently merged into the endosome membrane [45, 47]. Fe^3+^ in the acidic pH (5.5–6.0) of late endosome is released from a transferrin-TfR complex while, transferrin remained bound to TfR and reconverted to Apo-Tf. Besides, endosomal ferrireductase such as 6-transmembrane epithelial antigen of the prostate (Steap) reduces insoluble Fe^3+^ to soluble Fe^2+^ that is transported from the endosomal lumen into the cytosol by DMT1. Apo-Tf bound to TfR is recycled to the cell surface and dissociates from the receptor at a pH of 7.4 [38, 47–50]. Here, TfR is ready to bound the next Holo-Tf and initiating recycling [51]. Cytosolic iron confronts several paths: (I) participation in biological functions by embedding within metalloproteins, (II) participation in mitochondrial energy transduction, (III) storage in the form of ferritin [48, 52]. Besides, lysosomal degradation of ferritin leads to the formation of an iron-storage complex, namely, haemosiderin, that is related to pathophysiological states (e.g., iron overload) and involved in reactive free radical generation [30, 48]. Iron homeostasis is maintained by multiple factors such as hepcidin hormone and iron-regulatory proteins (IRP1 and IRP2)/iron-responsive element (IRE) signaling pathway [42]. Hepcidin, which is produced by the liver, is an essential systematic regulator. When iron is abundant, hepcidin binds to enterocyte ferroportin and blocks the export of iron out of the cell [35, 42]. At the cellular level, the IRP/IRE signaling pathway regulates iron homeostasis depending on the body’s iron concentrations. In iron deficiency, IRP binds to the IRE motif at the 5′-untranslated region (5′ UTR) of ferroportin and ferritin transcripts to suppress translation of their mRNAs. Whereas, binding of IRPs to the IRE motif at the 3′-UTR of TfR and DMT1 transcripts stabilizes their mRNAs to enhance translation. These processes lead to decreased plasma iron and increased cellular iron for use in the metabolic processes [50]. On the contrary, when the iron is abundant, IRP cannot bind to the IRE motif at 5'UTR of both ferroportin and ferritin transcripts and enhances translation of their mRNAs as well as IRP cannot bind to the IRE motif at 3′-UTR of TfR and DMT1 transcripts and destabilizes mRNAs to suppress translation [53].

## Iron in the brain

**Fig. 2:**
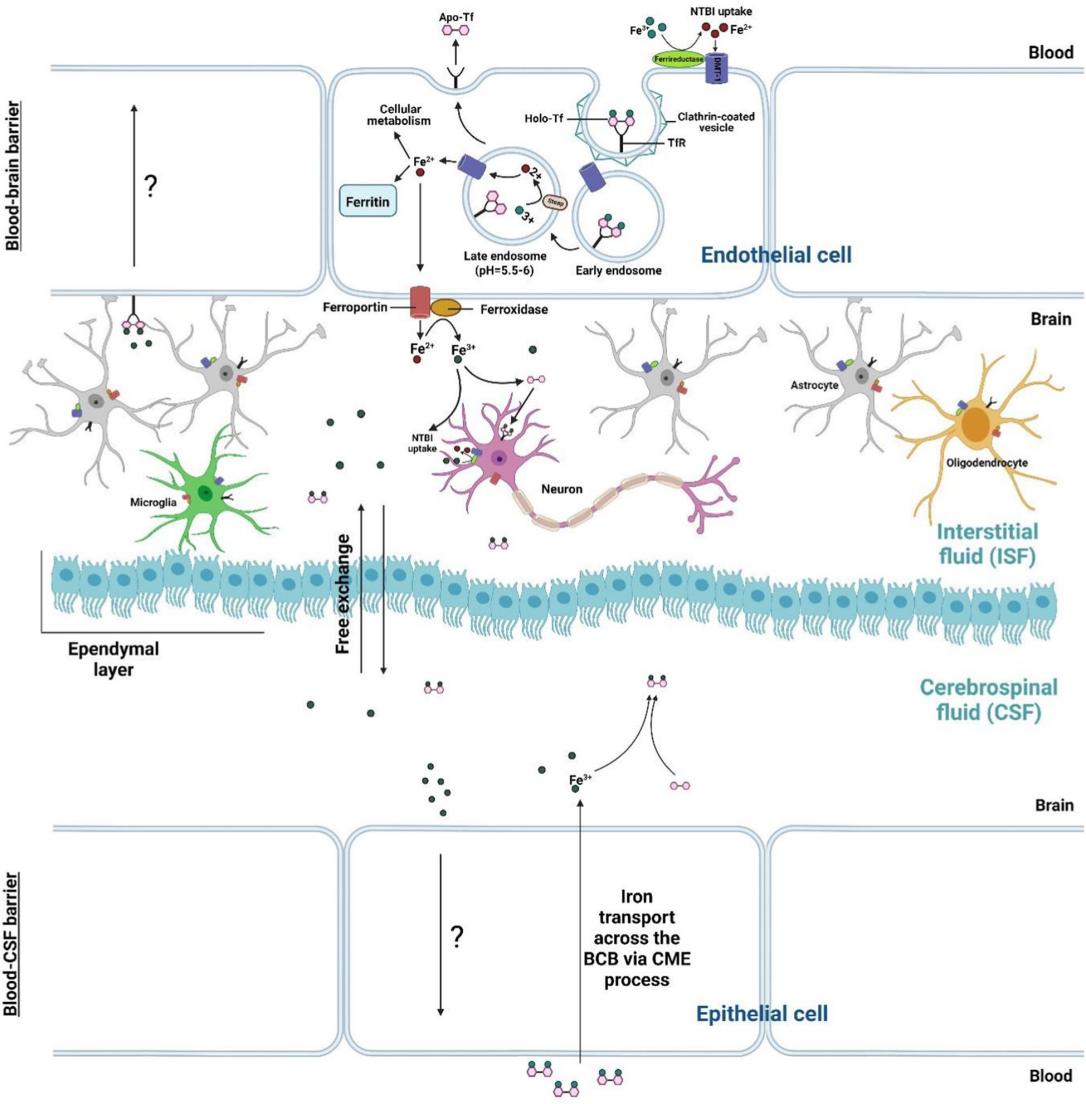
Entrance of iron into the brain: Holo-Tf binds to TfR on the membrane of the capillary endothelial cells of the BBB and choroid plexus epithelial cells of the BCB. The reduced form of iron can export from the membrane by ferroportin toward interstitial fluid and cerebrospinal fluid after dissociation from TfR. After re-oxidizing of Fe^2+^ to Fe^3+^ mediated by ferroxidases, Fe^3+^ binds to transferrin. Holo-Tf and free iron ions can freely exchange between CSF and ISF. There are ependymal cells between these two fluid compartments that are linked by gap junctions. Neural cells (e.g. oligodendrocytes, astrocytes, microglia, and neurons) uptake Holo-Tf via receptor-mediated endocytosis in ISF. There are several mechanisms for iron recycle to the systemic circulation. For example, Holo-Tf binding to TfR on the abluminal membrane of BBB and iron reabsorption into the blood plasma which is triggered from subarachnoid and transporting through BCB. However, the exact mechanism of iron export back to the systemic circulation is not clear. Holo-Tf, Holo-transferrin; TfR, transferrin receptor; Apo-Tf, apo-transferrin; DMT1, Divalent metal transporter 1; NTBI, non-transferrin bound iron; BBB, blood–brain barrier; BCB, blood-CSF barrier; CSF, cerebrospinal fluid; ISF, interstitial fluid; CME, clathrin-mediated endocytosis. This Figure was created by BioRender (https://biorender.com/)

Due to the significant relevance between neurodegenerative diseases and abnormal iron metabolism, an accurate description of the fate of iron in the CNS is necessary [48]. Iron in CNS plays an essential role in many normal neural functions, including cell division, energy production, axons myelination, dendritic branching, and neurotransmitters synthesis such as dopamine and serotonin [24, 53–55]. Iron is a co-factor for tyrosine hydroxylase that is involved in dopamine synthesis and tryptophan hydroxylase that is involved in serotonin synthesis [54]. Dopamine is a type of catecholamine in the brain that can be released to certain areas of the hippocampus, probably the CA1 region, and enhances long-term potentiation (LTP) [56]. Iron deficiency is associated with decreased myelin synthesis, which is formed by myelinating glial cells i.e., oligodendrocytes, followed by consequences such as memory impairment [57]. Iron transport to brain cells by the blood–brain barrier (BBB) and the blood-CSF barrier (BCB). Most of the iron enters into brain interstitial fluid (ISF) by crossing BBB, and some iron enters into the cerebrospinal fluid (CSF) by crossing BCB within the choroid plexus [58]. The Holo-Tf-TfR pathway is one of the well-known routes of iron towards the brain [59]. Like other cell types mentioned above, circulating Holo-Tf binds to TfR on the membrane of the capillary endothelial cells of BBB and choroid plexus epithelial cells of BCB. This binding resulted in cell membrane budding along with the Holo-Tf-TfR complex through the CME process. The reduced form of iron can export from the brain capillary by ferroportin toward ISF and CSF after dissociation from TfR. After re-oxidizing of Fe^2+^ to Fe^3+^ mediated by ferroxidases, Fe^3+^ binds to transferrin and uptakes by neural cells (e.g., oligodendrocytes, astrocytes, microglia, and neurons) via the receptor-mediated endocytosis [23, 24, 58, 60–62]. However, some iron may uptake in the form of NTBI, likely by DMT1 [59] after reduction of Fe^3+^ to Fe^2+^ by ferrireductase [63] (Fig. 2). Iron uptake by neurons includes transferrin-bound iron and NTBI. Upregulation of TfR on neurons in the iron deficiency, suggesting extensive transferrin-bound iron uptake through this receptor [64]. Neurons and other cell types likely acquire NTBI through DMT1. However, the mechanism of NTBI uptake has not been precisely clarified [65]. Iron exporter in neurons is the same as ferroportin that is expressed all over the cell membrane. Ferritin as an iron-storage protein has also been found in some neurons [64] (e.g., dopaminergic neurons) [66]. Iron is also present in the synaptic space of neurons, which is released from the axon terminus [24]. There are several mechanisms for iron recycle to the systemic circulation. For example, Holo-Tf binding to TfR on the abluminal membrane of BBB, and arachnoid granulations-mediated transportation has been proposed as a mechanism to export iron from the brain into the circulation [67]. Excess iron caused by pathological or senescence conditions also back to the systemic circulation. Moos et al., by injection of transferrin radiolabeled with ^59^Fe and ^125^I into the lateral ventricles, proposed a major route of iron reabsorption into the blood plasma which is triggered from subarachnoid and transporting through BCB [68]. Furthermore, the clearance of cerebral apoptotic/necrotic cells under inflammatory conditions via phagocytosis contributes to the efflux of iron into blood plasma from the brain by phagocytes [64]. However, the exact mechanism of iron export back to the systemic circulation is unclear and requires more studies.

## Iron overload-induced neurotoxicity

**Fig. 3:**
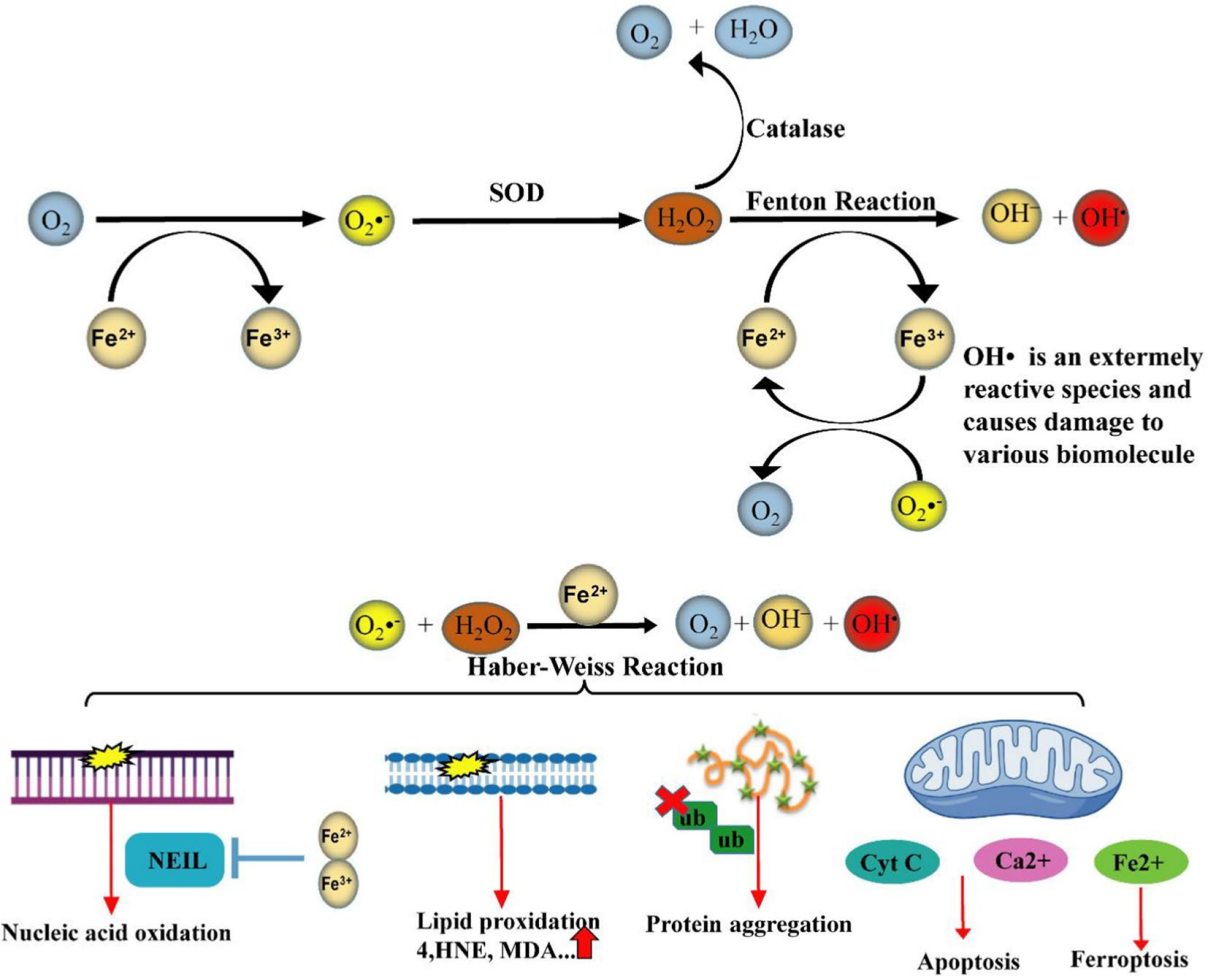
Cytotoxicity induced by iron overload: Oxygen (O_2_) reduction via Fe^2+^ produces Fe^3+^ and superoxide anion (O_2_•^−^). SOD enzyme converts the O_2_•^−^ to hydrogen peroxide (H_2_O_2_) and O_2_. H_2_O_2_ is converted to water (H_2_O) via antioxidant enzymes such as catalase. In the presence of redox-active bio metals such as free iron, Fenton reaction occur by reduction of H_2_O_2_ thereby Fe^3+^, hydroxide (HO^−^), and harmful hydroxyl radical (OH^•^) are produced. On other hand, Fe^3+^ reduction via O_2_•^−^ in the iron-sulfur proteins, renews Fe^2+^ for Fenton reaction. Accordingly, the reaction referred to the Haber–Weiss reaction which has required iron ions. Iron overload and ROS mutually reinforce each other and damage nucleic acids, lipids, proteins, and cellular compartments such as mitochondria. SOD, superoxide dismutase; HNE, hydroxynonenal; MDA, malondialdehyde; cyt C, cytochrome C; NEIL, nei like DNA glycosylase; ub, ubiquitination. This Figure was created by powerPoint and Adobe Illustrator

**Fig. 4:**
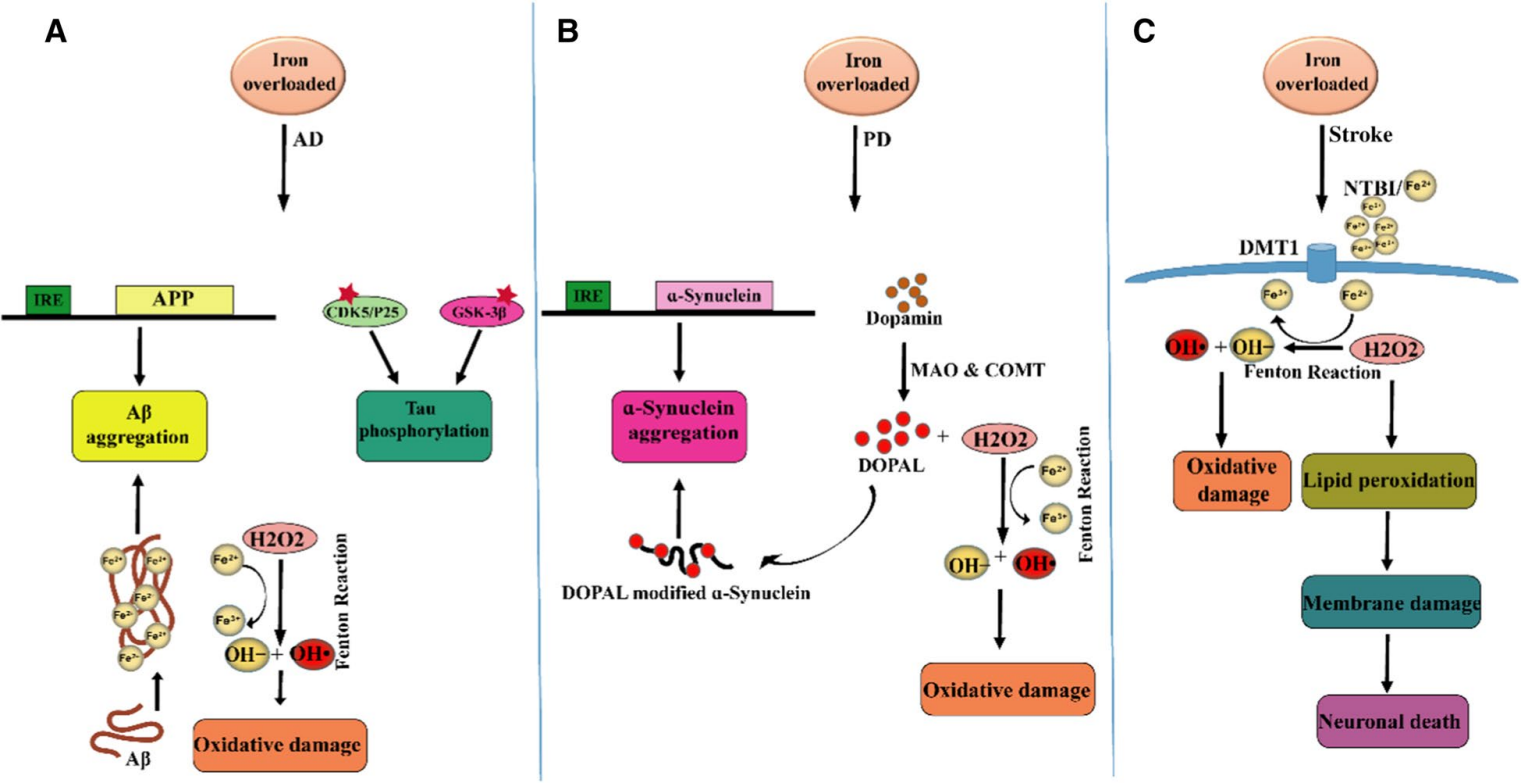
Correlation between iron overload and three dementia-associated diseases: **A** AD: iron overload causes Aβ aggregation by acting on the IRE site of APP mRNA and interaction with Aβ. Iron promotes the phosphorylation of tau by activating the CDK5/P25 complex and GSK-3β to form neurofibrillary tangles. The positive feedback loop among iron accumulation, oxidative stress, Aβ aggregation, and tau hyperphosphorylation causes neuron death. **B** PD: Within the synapse, dopamine can be broken down to DOPAL and inactivated by two enzymes including MAO and COMT. H2O2 is a normal product of monoamine oxidation via MAO. H2O2 can participate in the Fenton reaction and produce highly active free radicals. DOPAL can modify ɑ-Synuclein and lead to its aggregation. In addition, iron directly induces ɑ-Synuclein expression and aggregation. **C** Stroke: low oxygen condition caused by ischemic stroke leads to more iron influx into the brain. Acidic pH leads to dissociation of Fe^3+^ from transferrin and its reduction to Fe^2+^ thereby NTBI uptake occurs by neurons. Harmful oxidation products caused by Fenton/Haber–Weiss reaction induce neuron death. AD, Alzheimer’s disease; PD, Parkinson’s disease; CDK5, cyclin-dependent kinase; GSK-3β, glycogen synthase kinase-3β; MAO, monoamine oxidase; COMT, catechol-O-methyl transferase; IRE, iron-responsive element; APP, amyloid precursor protein; DOPAL, 3,4-dihydroxyphenylacetaldehyde. This Figure was created by powerPoint and Adobe Illustrator

Iron is a chemical element belonging to transition metals with electron donor and acceptor activity [69]. Despite iron is a crucial component in neuro functioning, its excess can lead to protein aggregation and oxidative stress. Its most destructive effect is neuronal cell death [14, 70]. Therefore, accurate regulation of iron homeostasis is required [69]. Iron accumulation mainly happens in normal aging but several age-dependent/independent factors are associated with its progression including smoking, high body mass index (BMI) [70], hereditary iron overload disorders (e.g. hemochromatosis) [71], transfusion-induced iron overload in types of anemia [72], and neurodegenerative diseases [73]. Besides, the usage of IONPs in the diagnosis and treatment of diseases (e.g. neurodegenerative disease) can result in iron accumulation [14, 15]. Excess iron is a critical player in reactions that damages tissue by overproducing ROS/RNS, which is briefly called RONS [74, 75]. This condition leads to an imbalance between antioxidants and prooxidants, which is referred to as nitrosative and/or oxidative stress [74]. Despite the relationship between iron overload and nitrosative stress, it is not sufficiently described. Therefore, in this study, we focus on the relationship between iron overload and oxidative stress. The brain is a sensitive organ to ROS due to continuous consumption of oxygen and iron, having a high percentage of polyunsaturated fatty acids (PUFAs) with high vulnerability to oxidation, and a weaker antioxidant defense in comparison with other tissues [76]. Under physiological conditions, ROS are produced as a result of cellular metabolisms. Oxygen (O_2_) reduction via Fe^2+^ produces Fe^3+^ and superoxide anion (O_2_•^−^) that is a precursor of other reactive species (2Fe^2+^  + 2O_2_ ↔ 2Fe^3+^  + 2O_2_•^−^). Superoxide dismutase (SOD) enzyme converts the O_2_•^−^ to hydrogen peroxide (H_2_O_2_) and O_2_ (2O_2_•^−^  + 2H^+^  → H_2_O_2_ + O_2_). H_2_O_2_ is converted to water (H_2_O) via antioxidant enzymes such as glutathione peroxidase (GPX) and catalase (CAT) (2H_2_O_2_ → 2H_2_O + O_2_). These reactions are carefully controlled and considered as a part of the cell signaling system [77, 78]. Nevertheless, H_2_O_2_ enters the destructive Fenton reaction in the presence of redox-active bio metals such as free iron. During the Fenton reaction, Fe^2+^ as an electron donor provides electrons for H_2_O_2_ reduction thereby Fe^3+^, hydroxide (HO^−^), and highly noxious hydroxyl radical (OH^•^) are produced (H_2_O_2_ + Fe^2+^  → Fe^3+^  + OH^−^  + OH^•^). On other hand, Fe^3+^ reduction via O_2_•^−^ in the iron-sulfur proteins, renews Fe^2+^ for Fenton reaction (Fe^3+^  + O_2_•^−^  → Fe^2+^  + O_2_) [77, 78]. Accordingly, the reaction referred to the Haber–Weiss reaction which has required iron ions (O_2_•^−^  + H_2_O_2_ → OH^•^ + O_2_ + OH^−^) [23]. Iron overload and ROS mutually reinforce each other and damage nucleic acids, lipids, proteins, and cellular compartments such as mitochondria [24]. ROS resulted from the Fenton reaction can lead to the oxidation of DNA bases. These lesions are repaired via a predominant mechanism of DNA repair called base excision repair (BER). But in the iron overload conditions, iron directly binds to two BER enzymes including nei like DNA glycosylase1 (NEIL1) and NEIL2 thereby inhibits their enzymatic activity [79]. Lipid peroxidation takes place under oxidative stress as well as the presence of iron. During lipid peroxidation, ROS directly reacts with membrane PUFAs to produce toxic aldehydes such as 4-Hydroxynonenal (4-HNE) and Malondialdehyde (MDA). Iron is an accelerator for this process. Furthermore, ROS by attacking membrane proteins leads to alteration in architecture, permeability, rigidity, and integrity of the membrane [76]. Lipid peroxidation products can produce misfolded proteins via carbonylation. The ubiquitin–proteasome system cannot degrade misfolded proteins thereby protein aggregation and neurodegeneration can occur [14]. The mitochondrial membrane is prone to damage due to a high level of PUFAs [80]. Excess iron-induced ROS increases mitochondrial membrane permeability, which releases iron from this organelle. Furthermore, excess iron impacts the cooperation of iron and calcium thereby downstream signaling pathways related to cognitive functions such as synaptic plasticity, mitochondrial function, and axon growth can be destroyed. Excess iron not only leads to mitochondrial dysfunction but also causes the release of calcium and cytochrome C from this organelle toward the cytosol and eventually cell death [14, 81]. Dopamine-induced neurotoxicity has also been reported as another mechanism of iron-dependent neurodegeneration. In this regard, metabolites resulting from excessive oxidation of dopamine (e.g., reactive quinones) cause neuronal death. This process is accelerated by excess iron and oxidative stress [82]. In physiological conditions, neurons remove oxidation products by several mechanisms. For example, glutathione (GSH) is a powerful antioxidant that balances intracellular oxidants level by binding to oxidation products and removing them from neurons [76, 82]. However, in pathological conditions, iron overload decreases the level of GSH that leads to TfR overexpression and re-induction of oxidative stress. A high level of TfR leads to more iron influx into the cell that exacerbates iron overload and oxidative stress [14]. Therefore, iron overload accompanied by primary oxidation products such as OH^•^, secondary oxidation products such as toxic aldehydes, and protein aggregation can induce neuronal cell death [76]. Ferroptosis is an iron-dependent cell death associated with degenerative and non-degenerative diseases such as Alzheimer’s disease (AD), Parkinson’s disease (PD), and stroke [81]. Ferroptosis is different from types of programmed and non-programmed cell death. It is the ultimate consequence of oxidative stress and lipid peroxidation (Fig. 3). During ferroptosis decrease in GSH level and GPX activity leads to lipid peroxidation in presence of Fe^2+^ [83]. Ferroptosis is prevented by antioxidants that are involved in iron chelation and anti-lipid peroxidation activity [81]. High concentrations of iron have been observed in various areas of the brain including the cerebral cortex, hippocampus, cerebellum, amygdala, and basal ganglia, in the healthy elderly, which these areas are most likely involved in neurodegenerative diseases. Iron concentration in the brains of patients with neurodegeneration is notably higher than in healthy aging [24]. Iron overload in aging can be caused by several pathological pathways including inflammatory conditions, increasing BBB permeability, and disturbance in iron homeostasis. Besides, iron overload in neuroglia and neurons aggravates neuroinflammation and leads to neuronal apoptosis [24]. There is a meaningful correlation between iron accumulation, normal brain aging, and neurological diseases such as AD [84], PD [85], and stroke [86] (Fig. 4).

## Iron in dementia-associated diseases

### Iron and Alzheimer’s disease

AD is a progressive brain disorder that slowly destroys learning, memory, and thinking skills. Age, gender, genetic susceptibility, lifestyle, and several pathological conditions such as diabetes and stroke as well as brain iron accumulation are risk factors related to AD [87, 88]. Senile plaques contain aggregates of extracellular amyloid-beta (Aβ) oligomers and neurofibrillary tangles (NFTs) contain aggregates of intracellular abnormal hyperphosphorylated tau protein are two common pathological hallmarks of AD. There is a relationship between iron accumulation and pathological hallmarks of AD. Abnormal levels of iron in the hippocampus and cortex of AD-affected subjects have been reported [75]. An in vivo study indicates iron deposits accompanied by senile plaques in the brain of a transgenic mouse model of AD by quantitative susceptibility mapping (QSM), a new technique in MRI [89]. The early plaques were formed in parallel with iron overload in a mouse model of AD [90]. Fe^3+^ within senile plaques can be converted to a more reactive form of iron, Fe^2+^, by Aβ [78]. On the other hand, 4-HNE raised from lipid peroxidation directly reacts with Aβ and produces oxidation products, which leads to Aβ aggregation [76]. Also, the Aβ peptide directly produces H_2_O_2_ in an iron reduction-dependent process, a process that exacerbates oxidative stress and iron overload [91]. Iron can increase the expression of amyloid precursor protein (APP) by affecting the IRE site of APP mRNA. Furthermore, iron can bind to Aβ and increase Aβ aggregation [92]. The relationship between iron deposition and tau phosphorylation has been demonstrated via cortical imaging by QSM and tau Positron Emission Tomography scanning (tau-PET) in AD subjects [93]. Iron promotes the phosphorylation of tau by activating the cyclin-dependent kinase (CDK5)/P25 complex and glycogen synthase kinase-3β (GSK-3β) to form NFTs and decrease the efflux of iron ions [92]. According to these explanations, it can be concluded that there is a positive feedback loop among iron accumulation, oxidative stress, Aβ aggregation, and tau hyperphosphorylation. Researchers could reduce the toxicity of the plaques, enhance the solubility of Aβ, and reduce the formation of NFTs by eliminating the iron ions by using iron chelators.

### Iron and Parkinson’s disease

PD is another neurodegenerative disease characterized by motor symptoms. Cognitive decline usually happens two decades before the diagnosis of motor symptoms. Hence, early diagnosis with considering cognitive decline can partly prevent the progression of PD [94]. PD occurs due to the degeneration of dopamine neurons particularly in a part of the substantia nigra called the pars compacta. Considerably, the loss of dopamine in the pars compacta disrupts voluntary motor control, increases the overall excitatory drive in the basal ganglia, and causes the characteristic symptoms of PD. Within the synapse, dopamine can be broken down and inactivated by two enzymes including monoamine oxidase (MAO) and catechol-O-methyl transferase (COMT) [95]. MAO activity is known to affect iron levels in animals and humans. There are complex interactions between free iron levels and MAO in the brain. However, increased oxidative stress appears to be a link between MAO, iron level, and neuronal damage. H_2_O_2_ is a normal product of monoamine oxidation via MAO. H_2_O_2_ can participate in the Fenton reaction and produce highly active free radicals. In aging, MAO and brain iron levels increase which leads to an increase in components of the Fenton reaction and damage of macromolecules [96]. Thus inhibition of MAO or removal of the Fe^2+^ ions by an iron chelator are two approaches with the same goal in PD patients at the same time, increases the monoamine levels, decreases components of the Fenton reaction, and the consequent oxidative stress.

Like AD, hyperphosphorylated tau and a decrease in soluble tau can cause iron overload in neurons via a decrease in APP-mediated iron export, which may be one of the causes of memory dysfunction in PD [97]. Besides, iron deposition was observed in structures supporting cognitive functions such as the hippocampus [85]. Evidence collected from 1988 to 2008 by A Jon Stoessl et al. showed abnormal deposition of iron, which is mainly together with ferritin in the substantia nigra neurons, motor-related area of PD patients. This data showed iron concentration is directly related to the severity of the disease [98]. Lewy bodies and Lewy neurites composed of abnormal ɑ-Synuclein filaments are the most important neuropathological characteristics of PD [94]. At the molecular level, there is a close relationship between ɑ-Synuclein aggregation and iron accumulation. Fe^3+^ from the Fenton reaction directly induces ɑ-Synuclein expression and aggregation. Overexpression of hepcidin, a potential regulator of iron transporters, reduces the accumulation of iron in the brain and Fenton reaction thereby ɑ-Synuclein aggregation and ROS production are reduced in the high-risk areas of the brain related to dementia and motor disorders [99, 100]. Thus the application of iron chelators that enhances the expression of hepcidin may inhibit ɑ-Synuclein aggregation.

### Iron and stroke

There is evidence for crosstalk between certain types of stroke, iron overload, and memory dysfunction [86, 101, 102]. Stroke is one of the major causes of memory dysfunction, and nearly 30% of stroke patients develop dementia within 1 year of stroke onset [103]. Atherosclerosis, diabetes, hypertension, smoking, high BMI, and dyslipidemia are risk factors for ischemic stroke [104]. Several mechanisms are involved in brain injuries induced by ischemia including inflammation, oxidative stress, the elevated concentration of intracellular calcium, enhanced excitatory amino acids, and increased levels of free iron and ferritin [105]. Post-stroke memory dysfunction can also be caused by vascular dementia, AD pathology [103], iron overload, and oxidative stress [86]. Edema formation by excess iron induces oxidative cell damage after a hemorrhagic stroke [106]. Iron deposition accompanied by a decrease in GSH and GPX and an increase in lipid peroxidation have been reported in neurons of ischemic stroke models [83]. Kondo et al. reported iron deposition in the hippocampus, striatum, and cerebral cortex in rats with transient forebrain ischemia. Late and early lipid peroxidation due to iron deposition after ischemia might be one of the causes of neuronal cell death [107]. Low oxygen condition caused by ischemic stroke leads to more iron influx into the brain. On other hand, acidic pH caused by ischemic stroke leads to dissociation of Fe^3+^ from transferrin and its reduction to Fe^2+^, thereby NTBI uptake occur. Neurons uptake NTBI and undergo Fenton/Haber–Weiss reaction, which produces harmful reactive radicals species and leads to lipid peroxidation and neuronal cell death [55].

## IONPs metabolism-induced neurotoxicity

IONPs consist of an iron oxide core and a protective coating [108, 109]. Iron oxides have several chemical structures such as magnetite (Fe_3_O_4_), maghemite (γ-Fe_2_O_3_), hematite (ɑ-Fe_2_O_3_), and wustite (FeO) [108]. Among them, Fe_3_O_4_ and γ-Fe_2_O_3_ are more widely used in nanomedicine [14]. Despite the great similarities between these two iron oxides, Fe_3_O_4_ is more magnetic and less stable than γ-Fe_2_O_3_ [110]. Bare IONPs accumulated upon entering the circulation due to hydrophobic interactions between themselves. IONPs accumulation stimulates the immune system thereby IONPs can be destroyed in an opsonization-dependent mechanism. Thus, a protective coating seems necessary for optimizing properties of IONPs including stability, biocompatibility, multi-functionalization, optimal biodegradation, hydrophilic interactions, and solubility [109]. Two types of IONPs are usually used for nanomedicine: superparamagnetic iron oxide nanoparticles (SPIONs) with a diameter of 50–100 nm and ultra-small superparamagnetic iron-oxide nanoparticles (USPIONs) with a diameter of up to 50 nm [111]. IONPs can enter the human body by many administration routes including intravenous (IV), intramuscular (IM), subcutaneous, intrathecal, intratumoral, oral, and nasal. Several mechanisms are proposed for IONPs uptake by cells such as passive diffusion, phagocytosis, and types of endocytosis whether dependent or independent from clathrin and caveolae [112]. The entrance route of IONPs into the cell depends on their physicochemical properties such as size, shape, type of coating, and functional group of these particles [113–115]. IONPs have a nanoscale size and high surface-to-mass ratio. Despite being an advantage, these properties can cause more reactivity and cytotoxicity [116]. Several studies have been performed on the possibility of IONPs toxicity in various tissues, especially neural cells. Despite being improving memory disorders, their relative role in neurodegeneration and exacerbating memory disorders have been somewhat discussed. Cytotoxicity of IONPs depends on physicochemical properties including size, shape, type of coating, surface charge, exposure time/concentration, functional groups, and also type of cell treated with IONPs [14, 117]. Besides, it has been reported that the oxidation state of Fe ions in the iron oxide core determines the cytotoxicity of IONPs. Fe_3_O_4_ due to high potential oxidation has shown more genotoxicity than γ-Fe_2_O_3_ in the A549 human lung epithelial cell [112]. Although, evidence from several studies suggests that IONPs contain Fe_3_O_4_ core had lower toxicity in comparison with γ-Fe_2_O_3_ due to their quick clearance from the body [14, 118]. In general, the major source of IONPs toxicity is the iron ions released from the core [119]. These iron ions along with other by-products of IONPs metabolism can interfere with iron homeostasis. In vivo studies indicated that liver ferritin levels enhanced after IONPs treatment, suggesting that IONPs are degraded, and their metabolic products induced alterations in iron responses [120, 121]. IONPs pass through the BBB by internalization mechanisms or destruction of endothelial cell membranes [14]. Iron uptake resulting from NPs metabolism depends on the levels of TfR expression on the cell surface [122]. IONPs have been reported to cross the BBB by interacting with the TfR on the abluminal membrane of endothelial cells. Also, BBB disruption and ROS enhancement caused by exposure to 10 μg/ml of Fe-NPs (10 and 30 nm) for 24 h in artificial BBBs have been reported [121]. In this regard, Jain et al. reported that IV administration of MNP (10 mg of Fe/kg in 100 µL of saline) in earlier time points did not change the levels of iron in the rat brain. Over time, binding of the released iron-transferrin complex to TfR on the BBB leads to an increase in iron content of the brain, especially one week after the MNP injection [122]. Thus, the level of TfR expression on the cell is another factor that differentiates NP uptake. Following the internalization of IONPs within the cell, they are placed in the acidic environment of the lysosome and metabolized resulting in the release of free iron ions into the cytosol. This degradation begins from the surface of NPs and gradually continues to their core. Released iron ions can participate in Fenton/Haber–Weiss reactions. The consequences of this event are manifested by the generation of early and secondary oxidation products that could damage cellular components such as nucleic acids, proteins, lipids, mitochondria [112, 123], and finally cause apoptosis [14, 124]. Thus, it is proven that CNS can be affected by IONPs. These conditions are somehow related to neurodegeneration [121]. During neurodegenerative diseases in which the BBB becomes permeable to many elements, especially NPs, the use of IONPs can exacerbate the disease [14]. There is evidence of NPs toxicity in dementia-associated diseases such as AD, PD [121], and stroke [125]. In vitro model of AD indicates iron oxide-based NPs can aggravate the condition by forming complexes with Aβ [126]. The c-Abl tyrosine kinase plays a key role in neuronal cell death in PD. The c-Abl activation, increased α-synuclein, reduced cellular proliferation, increased ROS, and mitochondrial permeability has been reported in neurons after SPIONs treatment by Imam et al. [121]. Leakage of electrons to the cytosol due to mitochondrial permeability causes a substantial reduction of striatal dopaminergic neurons in rats [121]. Iron depositions induced by IV injection of USPIONs [2 mmol iron/kg body weight (0.15 ml)] have been observed in the stroke mouse model. It has also been shown that USPIONs can access the brain parenchyma and CSF by crossing the BBB, which was found via detection of USPIONs in meningeal macrophages and phagocytes in CSF-bathed areas [125].

Iron concentration in the brain is not static and is affected by factors such as age, a poor iron diet, iron deficiency anemia, and iron overload disorders. The iron content of different regions of the brain varies. Macro divisionally the white matter has a higher concentration of iron. Local divisionally, globus pallidus, red nucleus, substantia nigra, caudate-putamen, and dentate nucleus have a higher concentration of iron [127]. Several studies have examined the tissue distribution of IONPs in the brain. Also, there is evidence for toxicity induced by coated IONPs. Frequent IV administration of ferumoxytol (8 mg/kg) as an iron replacement product for 4 weeks in rats showed that IONP can lead to iron accumulation in the ventricles. Iron concentration changes over time were quantified by the QSM technique. Slight changes in iron content in the striatum and corpus callosum were reported by using regions of interest (ROI) analysis, which may be related to iron deposition in the brain parenchyma. Also, the histopathological assessment showed choroid plexus hemosiderosis and midbrain vacuolation in the brain parenchyma [128].

In an in vivo study, radiolabeled aminopropyltriethoxysilane (APTS)-coated IONPs were instilled intranasally in Sprague Dawley rats in a concentration of 10 µg (in 10 µl). IONPs concentration in local areas on the seventh day of exposure was measure. The olfactory bulb, striatum, hippocampus, brain stem, cerebellum, and frontal cortex showed the highest concentration of IONP depositions, respectively. Even more than 50% of IONP remains in the striatum and hippocampus by 14 days later. Besides, oxidative damage increases in the striatum and hippocampus. Following in vivo study, toxicity mechanisms induced by IONP were investigated in dopaminergic neuronal PC12 cells. Incubated PC12 cells with IONPs (100 and 200 mg/ml) showed significant cytotoxicity including elevated MDA levels and a decrease in levels of GSH-PX and SOD. Exposed PC12 cells also showed an increase in phosphorylation of c-Jun, JNK, and p53, which were associated with oxidative stress and cell death [129]. To the best of our knowledge, there is no certain range of maximum permissible concentrations of IONPs in different areas of the brain. This varies for IONPs and depends on physicochemical properties and standardization.

## IONPs surface coating

It is well known that optimizing the physicochemical parameters of IONPs is highly effective to minimize the interactions between these NPs and cells, immune response, and toxicity. Whenever a new nanoparticle is made, one of the first important things that need to be considered is its surface coating. The coating preserves the inner core of the nanoparticle and prevents the release of nanoparticles. However, the coating itself should not be toxic. One way to reduce the toxicity of nanoparticles is to coat them. Coating nanoparticles, in addition to making them viable and reducing their toxicity, also makes them more efficient [6]. Depending on the type and application of nanoparticles, different types of coatings have been used. Some coatings are used to protect nanoparticles from possible changes in the gastrointestinal tract, and some are used to conjugate materials into nanoparticles. Nanoparticle coatings affect their absorption and biodistribution in the body and are even effective in the autophagy of nanoparticles [14, 108, 117]. Like most nanoparticles, IONPs contain an iron oxide core and a protective coating. The surface coating can optimize IONPs function and their cytotoxicity properties. Therefore, the surface coating seems essential for optimizing properties of IONPs including stability, biocompatibility, multi-functionalization, optimal biodegradation, hydrophilic interactions, and solubility [109]. The surface coating could be related to IONPs physicochemical characteristics including interactions with biological components, cellular uptake, in vivo fate, and toxicity. It also affects the fate and biological effects of IONPs. The coating provides an attachment layer to different molecular ligands such as chemical groups (e.g., carboxyl and hydroxyl) and biomolecules (e.g., peptides and polysaccharides), the so-called functionalization [6]. Because of colloidal instability of bare IONPs, several natural and synthetic surface coatings such as chitosan, dextran, citrate, Pluronic, polyethylene glycol (PEG), poly(ethylenimine) (PEI), polyvinyl alcohol (PVA), silica, and gold have been used. PEG is the most popular coating polymer because it prevents the aggregation and opsonization of nanoparticles. PEI is used to convey DNA/siRNA. In our studies, we have used dextran, a hydrophobic natural polymeric carbohydrate with a neutral charge [115, 130–134]. Although the proper coating can stabilize IONPs, avoid agglomeration, and prevent the dissolution and release of toxic ions, there are reports regarding the relative toxicity of surface-coated IONPs. In this regard, Kazemipour and et al. reported that 100 mg/kg of IONPs coated by dextran induced a significant decrease in hepatic GSH level and CAT activity and a significant increase in hepatic MDA level of rats [135]. In a study Feng, et al. showed that PEI-coated IONPs caused severe cytotoxicity through multiple mechanisms such as ROS production and apoptosis. Whereas, PEGylated IONPs showed a slightly cytotoxic effect only at high concentrations. In addition, PEI-coated IONPs exhibited dose-dependent lethal toxicity in BALB/c mice [136]. The results of an in vitro study showed that magnetic nanoparticles coated with the shortest 0.75 kDa polyethylenoxide (PEO) tails caused cytotoxicity and there was an inverse correlation between the PEO tail block length with toxicity [137]. Badman and et al. examined the dose-dependent neurotoxicity of dextran-coated IONPs on cultured primary neurons and showed that concentration above 20 µg/ml increased cellular ROS and lead to cell death [138]. Therefore the presence of a strong iron chelator can improve the potential benefits of IONPs with different coating and prevents the possible toxicity of them.

## FDA-approval commercial IONPs

There are a large number of nanoparticles that are in the final stages of development that their potential medical applications have been confirmed [136]. SPIONs are one of the few FDA-approved nanoparticles that are commonly used as a contrast agent for magnetic resonance imaging (MRI) and iron replacement therapies [139]. Many of IONPs have been under several clinical trials and some of them are approved by the European Commission (EC) and U.S. Food and Drug Administration (USFDA). It is estimated that the process of discovering a drug, pre-clinical studies including testing on animals and proving an effective and safe dose, clinical studies, and then FDA approving, takes about 10–15 years [140]. To the best of our knowledge, ferumoxide (Feridex I.V.), ferumoxsil (Lumirem), and ferumoxytol (Feraheme) are IONPs approved by the USFDA [141] (Table 1). Many of them were discontinued in the market because they were approved about 30 years ago [142]. For example, ferumoxide and ferumoxsil were withdrawn from the market in 2008 and 2009, respectively. Ferucarbotran (Resovist) and ferumoxtran-10 (Combidex) are two clinically approved SPION developed for contrast-enhanced MRI [143]. However, they have not yet been approved by USFDA 1^,^ 2. In addition, Nanotherm is an amino silane-coated SPIONs that was approved by Europe for glioblastoma multiforme (GBM) therapy in 2010, while in the US it is in late clinical trials and pending USFDA approval in 2021 [144]. There are several FDA-approved iron formulations such as INFeD (Dexferrum) and Venofer.

**Table 1:** USFDA-approval commercial IONPs

Generic name of SPION	Ferumoxide	Ferumoxsil	Ferumoxytol
Trade name	Feridex I.V (USA) Endorem (EU)	Lumirem (USA) GastroMARK (EU)	Feraheme (USA) Rienso (EU)
Approval date	USFDA approval in 1996 Discontinued in 2008	USFDA approval in 1996 Discontinued in 2012	USFDA approval in 2009
Coating	Dextran	Siloxane	Carboxymethyl-dextran
Size	120–180 nm	300 nm	20–50 nm
Blood half-life	10 min	NA	14 h
Recommended dose	30 μmol Fe kg^−1^	600 mL (105 mg Fe)	An initial 510 mg dose followed by a second 510 mg dose 3 to 8 days later
Administration route	Intravenously	Oral suspension	Intravenously
Application/indication	Visualization of liver tumors and metastasis	Contrast enhancement agent for MRI of gastrointestinal and examination of the bowel	Iron replacement therapy for the treatment of iron deficiency anemia in adult patients with chronic kidney disease
Human side Effects	Nausea, leg pain, headache, chest pain, hives, vasodilation	Nausea, vomiting, diarrhea, and cramps, iron overload, hiatal hernia	Hypotension, infusion site reactions, gastrointestinal complications, dizziness
Animals toxicology	Despite evidence of long-term toxicity, no iron overload, oxidative stress, pathological brain cell, and myelin changes were detected [298]	There are no carcinogens, genotoxicity, reproductive and developmental toxicity in vivo studies. No neurotoxic side effects have been reported (see Foot note link 14)	Repeat-dose toxicity, reduction in body weight gain and food consumption, enhancement in pigmentation intensity, decrease in fetal weights and external and soft tissue fetal malformations in vivo studies. No neurotoxic side effects have been reported (see Foot note link 15)

INFeD has been administrated intravenously (IV) or intramuscularly (IM) for iron-deficiency patients that oral administration is not effective for them^3^. The recommended dose of INFeD is 50 mg iron/ml as an injectable solution^4^. INFeD was teratogenic in animal models at a dose about 3 times the maximum anticipated dose of humans^5^. IM injection of iron dextran (100 mg of iron/kg) in divided doses over 12 weeks showed no abnormalities in rats. However, administration of 1000 mg of iron/kg causes enlargement of the liver and spleen of rats^6^. To the best of our knowledge, no neurotoxicity was reported for INFeD, remarkable research still needs to be done to ensure that neurotoxicity is negated.

Venofer (iron sucrose injection, USP) is another iron replacement product that is used for the treatment of anemia related to CKD intravenously. The initial US approval was in 2000 7. Venofer is available in different doses based on individual iron deficiency including 200 mg elemental iron/10 ml, 100 mg elemental iron/5 ml, and 50 mg elemental iron/2.5 ml^8^^,^ 9. Studies in rats and mice showed bleeding in the gastrointestinal tract and lungs, hypoactivity, pale eyes, and mortality after IV injection of iron sucrose at a dose about 3 times the maximum anticipated dose of humans (see Foot note link 9). However, we could not find reports show Venofer neurotoxicity.

Ferumoxytol is an iron replacement product that is made of carboxymethyl dextran-coated USPIONs and its trade name is feraheme [145]^10^. Ferumoxytol received FDA approval in 2009 and is used for the treatment of iron deficiency anemia in adult patients with CKD. The recommended dose of ferumoxytol is an initial 510 mg undiluted IV injection followed by a second 510 mg injection 3 to 8 days later. Ferumoxytol is injected at a rate of up to 1 ml/sec (30 mg/sec) (see Foot note link 10). Animal toxicology and pharmacology of ferumoxytol demonstrated a dose-dependent increase in plasma half-life. Among tissues, the liver, spleen, and central lymph node have the highest concentrations of ferumoxytol. Radiolabeled ferumoxytol (^59^Fe) was also observed in the red blood cell fraction during 24 h. Although carbohydrate coating has considerable excretion via urine and feces, radiolabeled ferumoxytol studies indicated iron of ferumoxytol has negligible renal clearance. Ferumoxytol injection up to 12 mg iron/kg/day for 13 weeks in rats (at a dose about 12 times the maximum anticipated dose of humans) and dogs (at a dose about 40 times the maximum anticipated dose of humans) showed a reduction in body weight gain and food consumption, and enhancement in pigmentation intensity, while clinical doses had no toxic effect on the immune system^11^. Animal studies showed no reproductive and developmental toxicity induced by ferumoxytol at daily doses of 31.6 mg Fe/kg during organogenesis for 12 days in rats. Excessive administration of ferumoxytol may cause excess iron storage accompanied by iatrogenic hemosiderosis. Thus, iron monitoring during treatment, especially in people with iron overload is necessary 12. Ferumoxytol is the only available IONPs for safe use in FDA-approved guidelines [146]. Currently, there are 31 clinical trials of Ferumoxytol for the treatment of iron deficiency anemia which 15 of them have completed^13^. Published reports have shown not only Ferumoxytol has not neurotoxic side effects, but also it can be used as a therapeutic agent in the central nervous system [147–153]. It has been shown that Ferumoxytol can be metabolized and is not deposited in the brain [147].

Green-synthesized magnetic iron oxide nanoparticles have a toxic effect on different brain regions and the effect varies according to the brain area [154]. A review study claims that ultrasmall superparamagnetic iron oxide nanoparticles as an emerging tool could be used for imaging of the brain while having a good safety profile [155]. A systemic review showed that the applications of the SPIONS for targeted delivery of drugs into the CNS had no significant toxicity [156]. As has been mentioned before surface coatings and particle size influence potential mechanisms of toxicity. Therefore, some SPION are safe for certain biomedical applications, while other applications need to be considered more carefully. In general, the available studies do not provide sufficient evidence to fully assess the potential risks for human health related to SPION exposure including USFDA approved. Further research regarding to SPION toxicity is needed [157].

In vivo studies results from acute toxicity, immunotoxicity, neurotoxicity, genotoxicity and reproductive toxicity researches in various animal models do not provide a clear overview on SPION safety yet, and epidemiological studies are almost inexistent. More investigation is needed to fully figure out how SPIONs interact with cells and what, if any, potentially adverse health outcomes can derive from SPION exposure [158, 159]. In the following sections, we describe quercetin (QC) could reduce the toxicity of SPION.

## Administration routes of IONPs

Oral, IV, local, and topical administration are human FDA-approved routes for the delivery of IONP. IV and oral are the most common routes that improve the therapeutic potential of nanoparticles [160]. Each of them has advantages and adverse effects and its choice depends on the target site, favorable application, and standardization [140]. Ferrous fumarate, ferrous, and, Ferumoxsil are administrated orally [143]. Oral administration is cost-effective, non-invasive, simple, and available for the general public but up to 50% of patients experience gastrointestinal complications [161]. Poor absorption, poor compliance, intestinal barrier, first-pass metabolism, gastrointestinal side effects, hepatotoxicity, and intact intestinal mucosa requirement for uptake are some of the adverse effects of oral iron administration [162, 163]. In addition, the bioavailability of ferric iron salts or ferric iron complexes is low that can prolong the duration of treatment [161]. Several NPs have prepared and standardized for IV administration. InFed as iron dextran, Venofer as iron sucrose, Injectafer as a ferric carboxymaltose and ferumoxide, ferucarbotran, ferumoxtran, and ferumoxytol as IONP are administrated via IV infusion [145]. IV iron administration is an alternative clinical treatment option for patients when oral iron is ineffective or not tolerated. IV iron formulations are increasingly safe, but there is still a risk of systematic toxicity, hypersensitivity reactions, anaphylaxis, hepatotoxicity, infusion reactions, and venous access and infusion monitoring requirement [139, 164]. A comparison between the risk of anaphylactic reactions related to IV iron products in 2015 showed all IV iron products are associated with anaphylaxis in patients in the US medicare nondialysis population [165]. The liver as a site for the first-pass metabolism is vulnerable to the toxicity of NP and has been shown to accumulate administered NP, even long after the end of the exposure. Therefore, hepatocellular toxicity is the main side effect of both oral and intravenously administration [139]. Gastrointestinal side effects due to the direct toxicity of ionic iron are the main side effect of oral iron [161]. The release of iron from the iron-carbohydrate structure and increase in transient concentrations of labile plasma iron is the hypothesis for the pathogenesis of acute oxidative stress induced by both oral and intravenously administration of iron oxide. Iron induces the Fenton chemistry and the Haber–Weiss reaction to promote the formation of highly reactive free radicals [142]. Iron toxicity is often dose-dependent and can be treated by gastric lavage with an iron chelator such as deferoxamine [164]. There are a large number of synthesized iron oxide nanovector to carry and deliver an antibody, siRNA, bioactive molecules, and drugs that suffer from limitations of the systemic circulation. The use of the IONP vector can reduce drug side effects by targeted delivery systems [166]. These studies show that SPIONs tend to be absorbed by liver macrophages [167]. For example, the result obtained from IV administration of SPIONs in mouse xenografts showed no significant cytotoxicity, except showed excess iron storage in the liver [168]. Another study investigated the effects of both the SPIONs coupled with anti-EGFR (Epidermal Growth Factor Receptor) antibody and aptamer in targeting breast cancer cells. The aptamers-bound SPIONs showed less damage and cytotoxicity, however, aggregation of SPIONs was the main problem [169]. In general, targeting specific areas by SPIONs leads to a high concentration of local iron. This can lead to impaired iron homeostasis, toxic implications in the exposed tissue, and pathological cellular reactions. Oxidative stress, epigenetic alterations, cytotoxicity, and inflammatory reactions are the possible adverse effects [167]. In the following sections, we suggested that the simultaneous application of QC in combination and especially conjugated form can be an effective strategy to reduce possible toxicity and aggregation of IONPs.

## Quercetin

QC is one of the most important defense compounds against foreign environmental agents in a variety of plants [18]. QC (3,3′,4′,5,7-pentahydroxyflavone) with a molecular weight of 302.236 g/mol and a molecular formula of C15H10O7 is a yellow powder/needles that is soluble in alcohol and glacial acetic acid and is insoluble in water. The chemical structure of QC is based on flavone-backbone (C6–C3–C6) and is made of three aromatic rings (A, C, and B). A and B are benzene rings that are joined through the C pyrone ring^14^ [17]. The unique structure of QC is dependent on the presence of 3-OH and 5-OH groups in A-C rings, 3′,4′-dihydroxy groups (catechol moiety) in B-ring, and double bonds. QC is widely distributed in fruits, vegetables, and beverages [170].

**Fig. 5:**
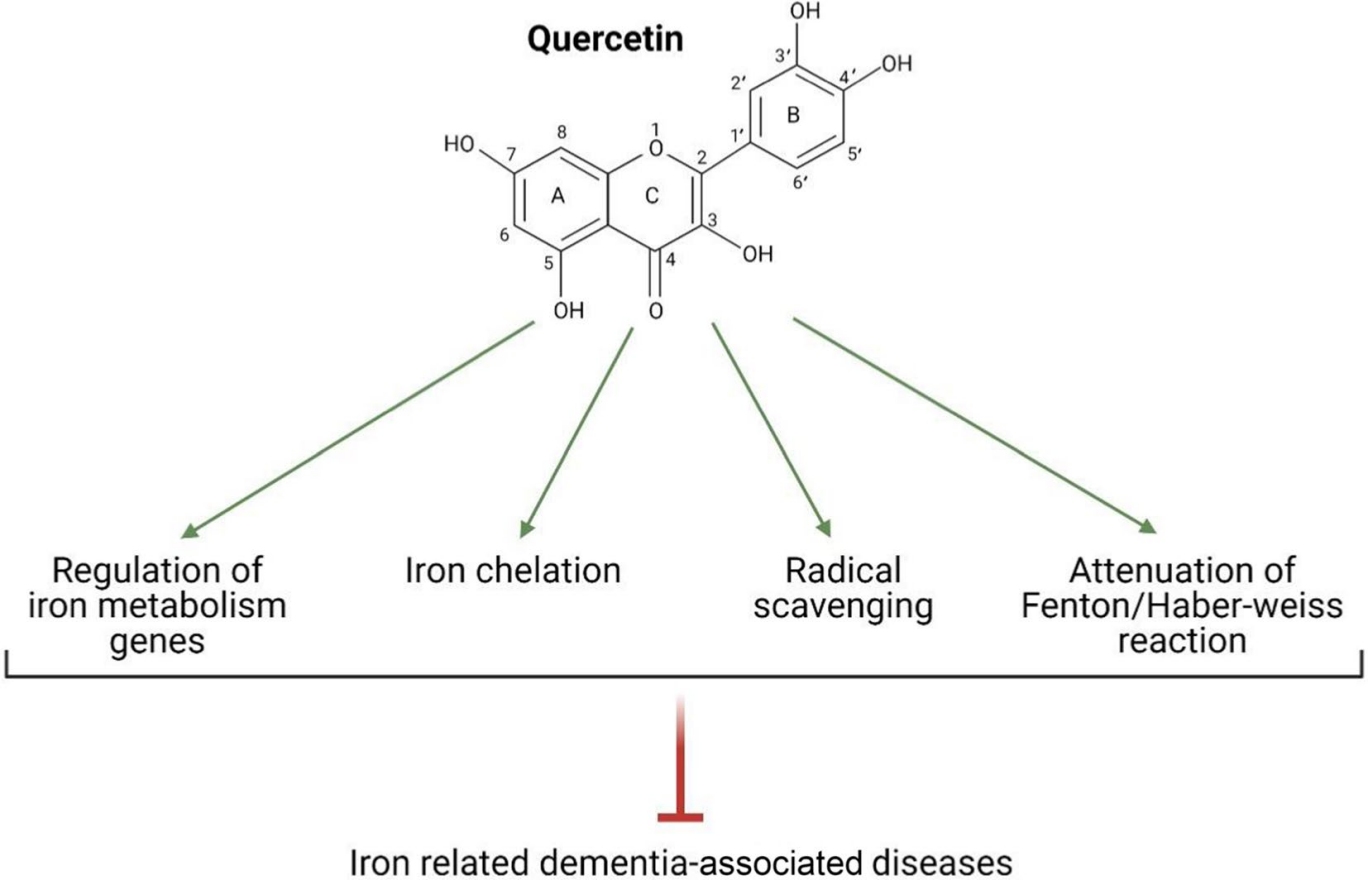
Underlying molecular mechanisms of quercetin (QC) against iron overload: QC directly exerts protective effects against iron overload via iron chelation and acts against iron overload via several mechanisms including binding QC to the free radicals, regulation of iron homeostasis genes, and regulation of enzymes involved in the Fenton/Haber–Weiss reaction. This Figure was created by BioRender (https://biorender.com/)

Glycosylated derivatives are the predominant forms of QC in plants. Sugar segments such as glucose and galactose can attach to OH groups, notably at position 3 and affecting the QC bioactivity and the quality of its uptake [171]. Glycosylated QC can be deglycosylated by bacteria derived from the mouth and intestine and the β-glycosidase enzyme in the intestinal brush border membrane thereby leads to the aglycone formation. Aglycone is the absorption form of QC. Absorption mainly occurs in the small intestine and a very small extent in the stomach. Due to the lipophilicity of the QC, it probably passes through enterocytes via passive diffusion [170, 172–174]. After absorption, aglycone and its metabolites are transported to the liver, and the resulting metabolic products such as methyl, glucuronide, and sulfate metabolites are distributed to different tissues via the bloodstream [170, 173]. The type of QC metabolites depends on the source. For example, after onions consumption, the major forms of QC metabolites observed in plasma are QC-3′-sulfate, QC-3-glucuronide, and QC-3-sulfate [173]. According to in vivo studies, after treatment with QC, it was found in the small intestine, kidneys, lungs, liver, and with much lower concentrations in the brain, heart, and spleen [175]. Plasma clearance of QC ranged from 11 to 28 h, and routes of its excretion are via urine and feces [171, 176]. QC is considered a valuable ingredient in the diet due to its wide range of pharmacological effects. Anti-inflammatory, antioxidant, anticancer, antiviral properties are only a part of the beneficial effects of QC [177]. These protective effects are related to the molecular structure of QC and confirm the relationship between the structure and function of flavonoids [178]. Several studies reported that QC prevents the accumulation of iron and its consequences [18, 20, 179, 180]. QC directly exerts protective effects against iron overload via iron chelation [18]. Besides, QC indirectly acts against iron overload via several mechanisms including binding QC to the free radicals [20, 181], regulation of iron homeostasis genes [179], and regulation of enzymes involved in the Fenton/Haber–Weiss reaction [177, 180] (Fig. 5).

Based on the above data, QC supplements can be used as a useful and safe compound. The recommended dose of QC is between 500–1000 mg/day for a short time in capsule and powder forms, and routes of administration are oral and IV. Administration in doses more than 1000 mg/day may cause mild symptoms such as headaches, stomach aches, and tingling sensations 15.

## The direct and indirect function of QC against iron overload

### QC as an iron chelator

**Fig. 6:**
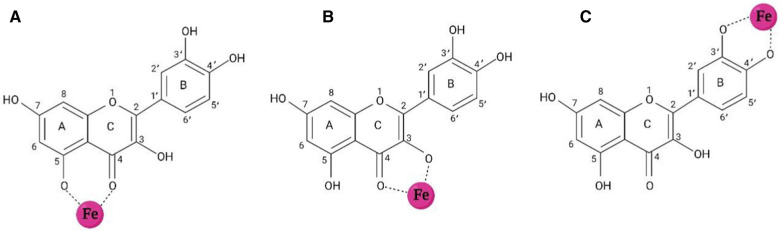
Iron-chelating sites of the quercetin (QC): QC can bind to iron ions via its own three sites including 3-hydroxy-4-oxo group in the **C** ring, 5-hydroxy-4-oxo group in **A** and **C** rings, and catechol moiety in the **B** ring to form QC-iron complexes. This Figure was created by BioRender (https://biorender.com/)

Iron chelation therapy refers to the elimination of excess iron to establish iron homeostasis in the body [182]. An ideal iron-chelating agent must have high-affinity iron-binding sites to form stable complexes with iron ions, not with other critical metal ions. Moreover, the consideration of optimal size, solubility, safety, and half-life of iron chelator to its penetrance in different tissues and avoiding accumulation is necessary [183–185]. QC is known as a strong phytochelator that can bind to both Fe^2+^ and Fe^3+^ [176]. Generally, there are three iron-chelating sites in the structure of QC including the 3-hydroxy-4-oxo group in the C ring, the 5-hydroxy-4-oxo group in A and C rings, and catechol moiety in the B ring. QC via its potential sites can bind to iron ions and form QC-iron complexes [182, 186–189] (Fig. 6). Types of metal ions and pH determine the preferred site of QC for metal-binding [190]. An in vitro study demonstrated that in the presence of extracellular iron, QC via binding to Fe ions keeps them in the extracellular compartment and prevents iron influx to the cell. In the presence of intracellular iron, QC permeates into the cell and traps Fe ions to prevent iron from entering the LIP [191]. Besides, QC can penetrate an iron overloaded cell and chelates excess Fe ions of LIP [192]. Altogether, iron participation in the Fenton reaction is suppressed [151]. Density functional theory (DFT) studies indicated QC can bind to Fe ions in the ratios of 1 Fe:1 QC, 1 Fe:2 QC, and 1 Fe:3 QC [18, 189]. Ren et al. reported that once a molecule of QC binds to the Fe atom, the preferred coordination sites for Fe are 3-hydroxy and 4-keto groups in the C ring, 5-hydroxy and 4-keto groups in the A and C rings, and catechol moiety in the B ring, respectively. 1:2 ratio (Fe: QC) is the most stable form of complex and 1:3 ratio (Fe: QC) causes saturation of Fe bonds and its neutralization [189]. Leopoldini et al. demonstrated QC in the forms of neutral and deprotonated can attach to Fe^2+^ and the most desirable configuration is 1:2 ratio (Fe^2+^:QC). Preferred coordination sites for Fe^2+^ are oxygen atoms belonging to 3-hydroxy and 4-keto groups in the C ring and 5-hydroxy and 4-keto groups in the A and C rings [18]. Also, the ability of QC to form complexes with Fe^3+^ has been demonstrated in ratios of 1:1 and 1:2 [151]. Afanas'ev et al. reported that the iron-chelating activity of QC inhibits the formation of hydroxyl or crypto-hydroxyl radicals resulted from the Fenton reaction [193]. Eman et al. reported a significant increase in brain iron levels in adult male albino rats following iron dextran injection. Also, a significant decrease in brain iron levels was observed following daily oral administration of QC. This decrease in iron levels is attributed to the chelating activity of QC [182]. Lesjak et al. reported that acute neutralization of iron by QC should be done via the chelating activity of this flavonoid because the application of 3-O-methyl QC (methylated QC in the 3-hydroxyl group) unlike intact QC didn’t decrease iron efflux into the plasma [194]. Guo et al. reported that Fe^2+^ chelating activity of QC is stronger than chromophoric Fe^2+^ chelator, ferrozine, at pH 7.2. Moreover, QC can compete with major cellular iron chelators such as ATP and citrate [195]. Vlachodimitropoulou et al. reported that QC in concentrations of less than 1 μM shuttles free iron ions from intracellular to extracellular space through GLUTs and prevents iron accumulation in the cells, in addition to acting as an iron chelator [196].

### QC as a free radical scavenger

Radical scavenging refers to the interaction between certain antioxidants/non-antioxidants and ROS or other reactive free radicals. Due to the high capacity of the molecular structure of QC, it can scavenge various RONS such as O_2_•^−^, OH^•^, ONOO^−^, and NO [20, 197], in which QC donates electron or hydrogen atoms from its own OH groups to free radicals [197, 198].

Three major mechanisms are considered for free radical scavenging by QC and other flavonoids: hydrogen atom transfer (HAT), sequential proton loss electron transfer (SPLET), and single electron transfer followed by proton transfer (SET-PT) [199, 200]. HAT mechanism is a single-step process and occurs through the transfer of a hydrogen atom from flavonoid to free radical. This process converts flavonoid to flavonoid radical (ArOH + R^•^ → ArO^•^ + RH). The OH bond dissociation enthalpy (BDE) is used to evaluation of antioxidant activity [201, 202]. In the SPLET mechanism, at the first step, the proton is removed from the flavonoid to form its anion (ArOH → ArO^−^  + H^+^). In the second step, electron transfer to radical leads to the formation of flavonoid radical and radical anion (ArO^−^  + R^•^ → ArO^•^ + R^−^), which is followed by protonation of the radical anion (R^−^  + H^+^  → RH) [202, 203]. Proton affinity (PA) in the first step and electron transfer enthalpy (ETE) in the second step is used to estimate the enthalpy of reactions [200, 202]. According to the SET-PT mechanism, the flavonoid is converted to its radical cation by donating a single electron to the radical (ArOH + R^•^ → ArOH^+**.**^ + R^−^). Radical anion reduction is governed by the deprotonation of flavonoid radical cation (ArOH^+**.**^ + R^−^  → ArO^•^ + RH) [202]. IP in the first step and proton dissociation enthalpy (PDE) in the second step are used for the estimation of enthalpies of SET-PT reactions [202]. The lower enthalpy value of these reactions is an indicator of the more desirable pathway for radical scavenging [178]. Li et al. reported SPLET mechanism probably is the main pathway of radical scavenging by QC [178]. The anti-radical activity of QC is affected by QC acidity [198] and free radical stability [200]. One study indicated proton affinity of QC’s OH groups is 3′-OH > 5-0H > 3-OH > 7-OH > 4′-OH, respectively [143], which suggests 4′-OH is the most acidic site, and it has a priority for proton loss during radical scavenging [180, 202]. Contrary, some studies proposed that 7-OH group is a more acidic site than the 4′-OH group, and it can more easily participate in radical scavenging [198, 204]. Another study showed OH groups in B and C rings have a high capacity to donate protons but OH groups in A ring participate in radical scavenging difficultly [200]. Thus, the most acidic site of QC has not been precisely determined.

QC inhibits the Fenton reaction via scavenging of O_2_•^−^ [205]. Also, ROS scavenging by QC leads to the formation of more stable and less reactive species [177]. In this process, QC donates electrons to reduce ROS and relatively neutralizes toxicity. For example, once QC interacts with OH^•^, the radical is converted to OH^−^,which is reduced into H_2_O by obtaining a proton [178]. QC suppresses lipid peroxidation via scavenging of lipid peroxyl radicals (ROO^−^) [205] via OH groups in A-C rings and catechol moiety in the B ring. When the OH groups of QC are methylated [206] or glycosylated [200], the inhibitory activity of QC is significantly reduced [200, 206].

### QC as a regulator of iron metabolism genes

Flavonoids can affect the expression of genes and the activity of proteins involved in iron metabolism [21]. QC potentially increases liver hepcidin expression [179]. This increase in expression is mediated by Nrf2 upregulation [207]. Nrf2 is a basic transcription factor that responses against iron overload via the regulation of iron metabolism genes such as hepcidin [207]. Sarkar et al. reported QC enhanced expression of Nrf2 protein in hepatocytes treated with NPs contain Fe_2_O_3_ that protects cells against death [208]. Ebrahimpour et al. reported QC increased expression of Nrf2 in the hippocampus of diabetic rats. It can be one of the factors that improve memory impairment induced by diabetes [209]. In the iron overload, ROS resulted from excess iron dissociates Nrf2 from its repressor (Keap1), and causing translocation of Nrf2 to the nucleus resulting in stimulation of BMP6 expression. BMP6-SMAD signaling pathway induces hepcidin expression in hepatocytes [207]. Hepcidin binds to intestinal iron exporter ferroportin, which leads to ferroportin endocytosis and its lysosomal proteolysis [179]. Hepcidin also downregulates TfR1 and DMT1 [210]. These mechanisms prevent excess iron entry to circulation and maintain iron homeostasis [179]. Thus QC prevents iron overload by regulating hepcidin through the BMP6-SMAD signaling pathway.

Du et al. indicated intracerebroventricular pretreatment with ad-hepcidin in iron overloaded rats reduced iron contents in the hippocampus, cortex, striatum, and substantia nigra. This reduction is mediated by reduced Tf-iron influx into the brain through BBB. Also, ad-hepcidin reduced expression of iron influx and efflux proteins (e.g., TfR, DMT1, and ferroportin) in cultures models of cerebral capillary endothelial cells and neurons, similar to the action of hepcidin in the intestine [182]. Lesjak et al. studied longer-term effects of QC in Caco-2 cells. Dual-luciferase reporter assays revealed the ferroportin-3′UTR has a target site for miR-17-3p. Exposure of these transfected cells to QC significantly decreased reporter activity. Thus QC with upregulation of miRNA can inhibit ferroportin expression and regulates iron homeostasis [194].

### QC as a regulator of enzymes involved in the Fenton/Haber–Weiss reactions

QC has antioxidant effects to inhibit the cascade of reactions that generate primary and secondary oxidation products. During oxidative stress conditions, enzymatic antioxidants such as SOD, GPX, CAT, and non-enzymatic antioxidants such as GSH, which are involved in Fenton/Haber–Weiss reaction and lipid peroxidation can be overwhelmed [211, 212]. As mentioned earlier, SOD, CAT, and GPX convert O_2_•^−^ and H_2_O_2_ to non-toxic H_2_O [78] and prevent Fenton/Haber–Weiss reaction and lipid peroxidation. GSH plays a vital role in the activity of antioxidants. Moreover, GSH directly can reduce OH^•^ and other reactive radicals to H_2_O and species with low reactivity [213, 214]. Nrf2 plays a regulatory role in the upregulation of antioxidant enzymes by binding to ARE in promoters of GSH-dependent antioxidant genes [215]. In the absence of these antioxidant defenses, cellular components are attacked by primary and secondary oxidation products [216]. Thus the application of exogenous antioxidant inducers is an ideal strategy for preventing oxidative stress.

The previous study indicates QC increases transcripts of CAT, SOD1, and Nrf2. Moreover, QC significantly decreases total antioxidant capacity (TAC) in the hippocampus of diabetic rats [154]. QC can directly interact with GPX and promotes enzyme activity by structural changes.

Moreover, QC binds to heme moiety or specific residues of CAT and enhances antioxidant activity [217]. QC induces antioxidant defense to eliminate oxidation products and restores oxidative balance [181]. Dong et al. reported that QC can increase intracellular GSH levels that are mediated by overexpression of glutamate-cysteine ligase catalytic subunit (GCLC), the first rate-limiting enzyme of GSH synthesis, in Caco-2 cell model exposed to H_2_O_2_ [218]. Kobori et al. reported that both chronic and high intake of QC reduced lipid peroxidation markers (e.g., MDA) and increased antioxidant enzymes such as GPX, SOD1, and CAT in the liver and adipose tissues in mice [219]. QC protects hippocampal neuronal cell line HT-22 of mouse against glutamate-induced neurotoxicity by promoting intracellular GSH levels, reducing Ca^2+^ influx, and ROS [220]. Interestingly, the complexation of QC with transition metal ions may exhibit SOD-like activity [221]. Therefore, QC could be a promising candidate for reducing oxidative stress.

## QC against dementia-associated diseases

### QC and Alzheimer’s disease

Anti-Alzheimer's effects of QC have been proven in various in vitro and in vivo studies [177].

QC modulates signaling pathways associated with AD such as PI3K/Akt, JNK/JUN, and Nrf-2-ARE pathways [16]. Moreover, QC interacts with enzymes engaged in the generation of Aβ plaques and NFTs [16]. Maria et al. reported that QC improves cognitive and behavioral skills in the aged triple transgenic AD mice model. QC decreases intracellular NFTs and extracellular deposition of Aβ peptides in the hippocampus and the amygdala in these mice [222]. One of the underlying mechanisms mediated by QC is the interaction of QC and acetylcholinesterase (AChE). AChE is an enzyme that hydrolyses acetylcholine (ACh) in the central and peripheral nervous systems. In AD subjects, the enzyme can promote the aggregation of Aβ peptides. Moreover, co-localization of AChE within amyloid deposits has been shown. Hydrogen-atoms from OH groups of QC bind to active site residues of AChE through hydrogen bonds and inhibits AChE thereby augments ACh levels in the space between pre-and postsynaptic neurons [16]. Another underlying mechanism mediated by QC illustrated by Shimmyo et al. QC treatment (20 μM) induces a remarkable reduction in Aβ (1–40, 1–42) levels by inhibition of β-secretase (BACE-1), the rate-limiting enzyme for Aβ production in neuronal cell culture. QC attaches to catalytic residues of BACE-1 including Asp32, Gln73, and Trp198 by C3-OH in the C ring, C7-OH in A ring, and both C4′ and C5′-OH in B ring, respectively [223]. Inflammation and apoptosis of neurons are other causes of neurodegeneration. The anti-inflammatory and anti-apoptotic activities of QC was studied by Khan et al. Intraperitoneal (i.p) injection of QC (30 mg/kg/day) increases PSD-95, a synaptic protein that is involved in memory performance, attenuates inflammatory responses by suppression of TLR4/MyD88/NF-κB signaling pathway and expression of inflammatory markers such as TNF-α, COX-2, NOS-2, and IL-1b. Also, QC treatment prevents mitochondrial apoptotic pathway by regulating Bax/Bcl2 ratio, Cyt c, caspase-3, and PARP-1 in the hippocampus and cortex of LPS-treated mice [224]. Liu et al. reported that the application of QC-modified sulfur NPs embedded into microbubbles under ultrasound treatment effectively reverses memory and learning disability via a reduction in apoptosis of neurons, inflammation, oxidative stress, and maintaining Ca^2+^ homeostasis [225]. Pretreatment of primary hippocampal cultures with QC one hour before induction of toxicity by Aβ (1–42) treatment, showed dose-dependent neuroprotective effects of QC including a significant reduction in lipid peroxidation, neurotoxicity, oxidative stress, and apoptosis. QC pretreatment (5 and 10 μM) remarkably decreased 4-HNE levels in Aβ1–42-treated neuronal cultures [226]. Therefore, QC can be considered as an effective phytocompound for the prevention of AD.

### QC and Parkinson’s disease

QC can be considered as a pharmacological agent against PD by different molecular pathways. QC can form QC-α-synuclein adducts in a 1:1 ratio by covalent binding to α-synuclein. Adducts attach to α-synuclein peptides to inhibit protein fibrillation [227]. QC treatment in a rat model of PD adverse cognitive dysfunction induced by 6-Hydroxydopamine injection. Its potential mechanism is probably mediated by increased activity of SOD, CAT, and GPX and a significant reduction in MDA levels and AChE activity in the hippocampus [228]. The i.p injection of QC (30 mg/kg) in the 6-hydroxydopamine-induced rat model of PD significantly increases GSH levels, decreases oxidative stress markers such as lipid hydroperoxides and protein carbonyl contents, and maintains neuronal survival in the striatum [229]. Treatment with QC protects cell culture and MitoPark transgenic mouse models of PD against 6-OHDA-induced neurotoxicity and promotes mitochondrial biogenesis. Besides, QC causes neuronal survival by activating PKD1, Akt, and downstream signaling pathways [230]. Neurotrophic effects of Akt have been shown in murine models of PD. In dopaminergic neurons, activation of Akt prevents apoptosis and preserves both neuronal viability and functionality [231]. QC enhances the activity of mitochondrial complex I, the largest and first enzyme of the electron transport chain that is defected in parts of the brain of PD patients. QC also scavenges hydroxyl radicals and improves mitochondrial function in the rotenone-induced rat model of PD [232]. Anti-inflammatory activity of QC studied in zebrafish models of PD. QC treatment reduced transcript levels of cytokines involved in neuroinflammation such as IL-1β, TNF-α, and COX-2 [233]. QC pretreatment (0.1 µM) attenuates apoptosis by enhancing the expression of Bcl-2 mRNA and reducing protein expression of the Bax/Bcl-2 ratio. Moreover, QC attenuates caspase-independent cell death by reducing nuclear translocation of AIF in MPP^+^-induced PC12 cytotoxicity [19]. Therefore, QC improves PD and preserves dopaminergic neurons by suppression of inflammation, apoptosis, oxidative stress, activation of cell survival pathways, and α-synuclein disaggregation.

### QC and stroke

Anti-ischemic activities of QC have been proven in several studies [234–237]. Dietary consumption of QC is associated with a reduced risk of stroke [173]. QC treatment in neonatal rats with hypoxia–ischemia-induced brain injury improves spatial learning and memory via increased myelin basic protein (MBP) expression that is responsible for myelination [238]. Pretreatment with i.p injection of QC (100 mg/kg) adverse ischemia/reperfusion-induced cognitive dysfunction in a mouse model via promoting Akt signaling pathway and subsequently inhibiting apoptosis induced by ASK1/JNK3/caspase-3 [239]. Antioxidant effects of QC pretreatment showed by Chen et al. QC significantly enhanced expression of Cu/ZnSOD, MnSOD, GPX, and CAT and reduced damage resulted from transient cerebral ischemic in hippocampal CA1 pyramidal neurons of gerbils. Antioxidant enzymes counteract oxidation products after an ischemic attack [240]. Nrf2 activation induced by QC can detoxify the cerebral microenvironment injured by stroke. Detoxification occurs via upregulation of antioxidants and anti-inflammatory capacity [241]. Treatment with Nrf2 activator in models of intracerebral hemorrhage, a type of hemorrhagic stroke, reduced neural damage. Nrf2 exerts antioxidative effects via suppression of oxidative stress and induction of antioxidant enzymes such as CAT, SOD, glutathione S-transferase [242]. The i.p injection of QC (50 mg/kg) markedly decreases MDA levels and both expression and activity of caspase-3. Moreover, QC significantly increases the activity of CuZn-SOD and GPX and ameliorates oxidative stress that is induced after subarachnoid hemorrhage, an uncommon cause of stroke, in the rat model [214]. QC treatment (50 mg/kg) can reduce markers of inflammation such as IL-1β, IL-4, IL-6, and TNF-α and ameliorate neuronal defects in an intracerebral hemorrhage rat model [243]. Pretreatment with oral QC (5 and 10 mg/kg/day) decreases ROS production and apoptosis via enhancing anti-apoptotic genes such as Bcl-2, Bcl-xL, and preventing caspase-3 cleavage in a rat model of cerebral ischemia/reperfusion [244]. Post-stroke disruption of BBB has been reported, which has consequences such as increase permeability, immune-inflammatory responses, neural damage, and cognitive dysfunction [245, 246]. QC treatment (25 μmol/kg) improves the structure of BBB and ameliorates its dysfunction. A possible mechanism is considered through activating the canonical Wnt/β-catenin signaling pathway in the rat cerebral ischemia/reperfusion model [247].

## Cooperation of QC and IONPs in memory enhancement

Despite the beneficial effects of QC, there are limitations for this flavonoid such as low water solubility, low absorption rate notably through the BBB, vulnerability to enzymatic reactions, quick metabolism, short half-life in the body, and rapid elimination from the circulation [248, 249]. In recent years, the application of QC in conjugation and combination forms with IONPs is considered a privileged approach to overcoming limitations. [181]. Several studies used IONPs combined with QC and showed QC attenuates toxicity induced by IONPs. In this regard, Katebi and colleagues showed significant cytotoxicity of IONPs and QC in a concentration above 100 μg/ml. Surprisingly, the treatment of PC12 cells with IONPs combined with QC caused a remarkable outgrowth of neurite and enhanced the neuronal branching complexity without any toxicity [250]. Another study showed that incubation of hepatocytes with 250 μg/ml IONPs decreased the cell viability and antioxidant ability. Incubation of hepatocytes with QC (50 μmol/l) 1 h before of IONPs exposure protects the cells from cytotoxicity [208]. An in vivo study showed that treatment with IONPs (50 mg/kg) dysregulates markers related to oxidative stress and apoptosis such as MDA, GSH, GSSG, AchE levels, and peroxisome proliferator-activated receptor-γ coactivator 1-α (PGC-1α), caspase-3, Bcl-2 expression levels in the brain tissue of rats. However, QC (100 mg/kg) adverse dysregulation of the above-mentioned biomarkers and attenuates oxidative damage and apoptosis raised from IONPs metabolism [15].

Application of the QC conjugated with superparamagnetic iron oxide nanoparticles (QCSPIONs) in animal models and cell culture has led to considerable results in our previous studies. The coprecipitation method was used to synthesize dextran-coated SPION and QC was loaded on these nanoparticles by appropriate linkers to produce QCSPION. Coprecipitation is one of the chemical-based synthesis methods frequently used in the literature. It is a simple, most effective, cost-effective, reproducible, durable, fast process that is easily transposable for industrial applications on a larger scale. This method provides a nanoscale material with high purity through an eco-friendly route, without dangerous organic solvents requirements, nor treatments under high temperature or pressure [251]. Coprecipitation can provide factors that enhance the efficiency of IONPs including nanoscale size, controlled shape, high magnetic susceptibility, the property of superparamagnetic crystal suspension, tailored surface chemistry for specific biomedical applications [252].

QCSPIONs are dextran-coated SPIONs that were synthesized by our teams. The chemical coprecipitation method was used to synthesize dextran-coated SPION. The nanoparticles were spherical and had diameters in the range of 30–50 nm. QCSPION nanoparticles were prepared by conjugation of QC to dextran-coated Fe_3_O_4_ nanoparticles by suitable linkers. As mentioned above coprecipitation is a simple, most effective, cost-effective, and fast process method that is easily transposable for industrial applications on a larger scale. QCSPION can be designed and translated reaction rates to a scale-up of the process. Several cellular and animal studies associated with the efficiency and cytotoxicity of these IONPs have been done. QCIONPs can be administered orally due to the release rate of QC from NPs [130]. The characterization result showed that 23% of the drug was released from QCIONPs during 4 h. This was progressively amplified and reach a maximum value of 61% during 8 h [130]. Therefore, oral administration of QCIONP provides enough time to homing IONPs in brain tissue and reach maximum efficiency of QCIONPs in comparison with IV injection. We should say that more studies are needed to increase QCIONPs effectiveness. Although at this time they have a long way away for humans, we hope that this review opens a new window for its clinical application in future.

In a study on H2O2-induced toxicity in PC12 cells, we reported the antitoxic activity such as the catalase-like activity, anti-inflammatory, and anti-apoptotic effects of QCSPIONs against the cytotoxicity of H2O2 [248]. In an in vivo study, we applied QC conjugated with IONPs (QCIONPs) to develop its brain distribution. We showed that the concentration of QC in the brains of QCIONPs-treated healthy rats was about 4.8 times for 50 mg/kg of QC and 8.6 times for 100 mg/kg of QC higher than rats treated with pure QC [118]. Therefore, it can be concluded that IONPs improve the bioavailability of QC and its passage through BBB. Besides, we reported that 100 mg/kg IONPs result in a remarkable reduction in renal CAT activity, hepatic GSH and CAT activity, and a significant enhancement in hepatic MDA in healthy rats. However, QC in conjugated form (50 mg/kg and 100 mg/kg) was able to neutralize these cytotoxic effects, so that hepatic TAC, GSH, MDA levels, and CAT activity did not show a significant difference between the QCSPION and the control groups [135]. In another study, we showed that treatment with QCSPIONs (50 and 100 mg/kg) during one week improved memory performance in healthy rats better than pure QC via their interaction with proteins involved in Long-Term Potentiation (LTP) [117]. Because diabetes plays a causative role in CNS-related diseases particularly cognitive dysfunction and dementia, we used QCSPIONs to improve learning and memory impairment in diabetic rats. We showed that oral delivery of QCSPIONs (25 mg/kg) during 40 days ameliorates learning and memory impairment of diabetic rats without any toxicity on blood glucose levels, body weight, and histological parameters [249]. In three separate studies, we focused on inflammation, oxidative stress, and glucose homeostasis as the underlying molecular mechanisms and some of the classical targets of QCSPIONs in diabetic conditions. We reported that QCSPIONs could improve cognitive dysfunction via targeting NF-κB/miR-146a, Nrf2/miR-27a, and GLUTs/miR-29 signaling pathways [209, 253, 254]. In addition, we showed that oral application of QCSPIONs (25 mg/kg) during 42 consecutive days protects AlCl 3-induced neurotoxicity in a rat model of AD via targeting the APP/miR-101 pathway [255]. Overall, according to these results, we conclude that QC as an effective metal chelator can attenuate toxicity in conjugation and combination forms. A comparison between the results from the conjugation and the combination methods demonstrates the conjugation of QC on IONP is more efficient to reduce neurotoxicity than QC supplementation even at a lower dose. The most important reason for the higher efficiency of QC in the conjugated form is that QC needs a delivery system to show its maximum efficiency.

According to previous studies, we hypothesized two mechanisms for increased cerebral bioavailability of QC in conjugated form with IONPs in comparison with pure form [118, 209]. In the first mechanism, IONPs are a carrier that delivers QC to the brain. Although, they cannot cross the BBB. One of the possible reasons for its non-entry into the brain is the presence of BBB membrane proteins that are involved in drug effluxes such as multidrug resistance protein and P-glycoprotein [118]. In the second mechanism, QCIONPs can pass through the BBB and get into the CNS [209]. Based on studies on the transfer of IONPs to the brain, IONPs pass through the BBB via paracellular or intracellular pathways [223]. This is facilitated in neurodegenerative diseases due to BBB alteration [256]. QCIONPs can enter the neural cell in NPs properties-dependent manner [112, 257]. After internalization, QCIONPs are trapped in the lysosome. In the acidic pH of the lysosome, QCIONPs are decomposed and QC is released from NPs [249]. Fe ions released from the IONPs can influence the expression of genes involved in the storage and transport of iron such as ferritin and ferroportin [112]. Given this, excess iron raised from IONPs can be trapped by ferritin and transferred out of the cell through ferroportin [258, 259]. Iron can also cause oxidative stress through the Fenton reaction, damaging DNA, lipids, and proteins and eventually leading to cell death [121, 260]. Besides redox cycle Fe^3+^ ↔ Fe^2+^ in the lysosomes resulting in ROS production, lysosomal permeability, the release of lysosomal proteases, and membrane permeability of other organelles. Furthermore, Fe ions can penetrate mitochondria and the nucleus to promote oxidative stress and damage biomolecules [121, 260]. In the presence of IONPs, the antioxidant pathway can be affected. For example, a significant reduction in GSH levels and SOD activity has been observed in neural stem cells treated with SPIONs [261]. Based on the evidence, free iron ions released from IONPs can trigger apoptosis pathways in the exposed cells via depolarization of the cell membrane, disrupting membrane potential, upregulation of Bax and Bad, downregulation of Bcl-2, and induction of caspase-3 activity [14]. Several studies have shown crosstalk between protein aggregation and iron overload-induced by IONPs metabolism [14, 121, 126].

**Fig. 7:**
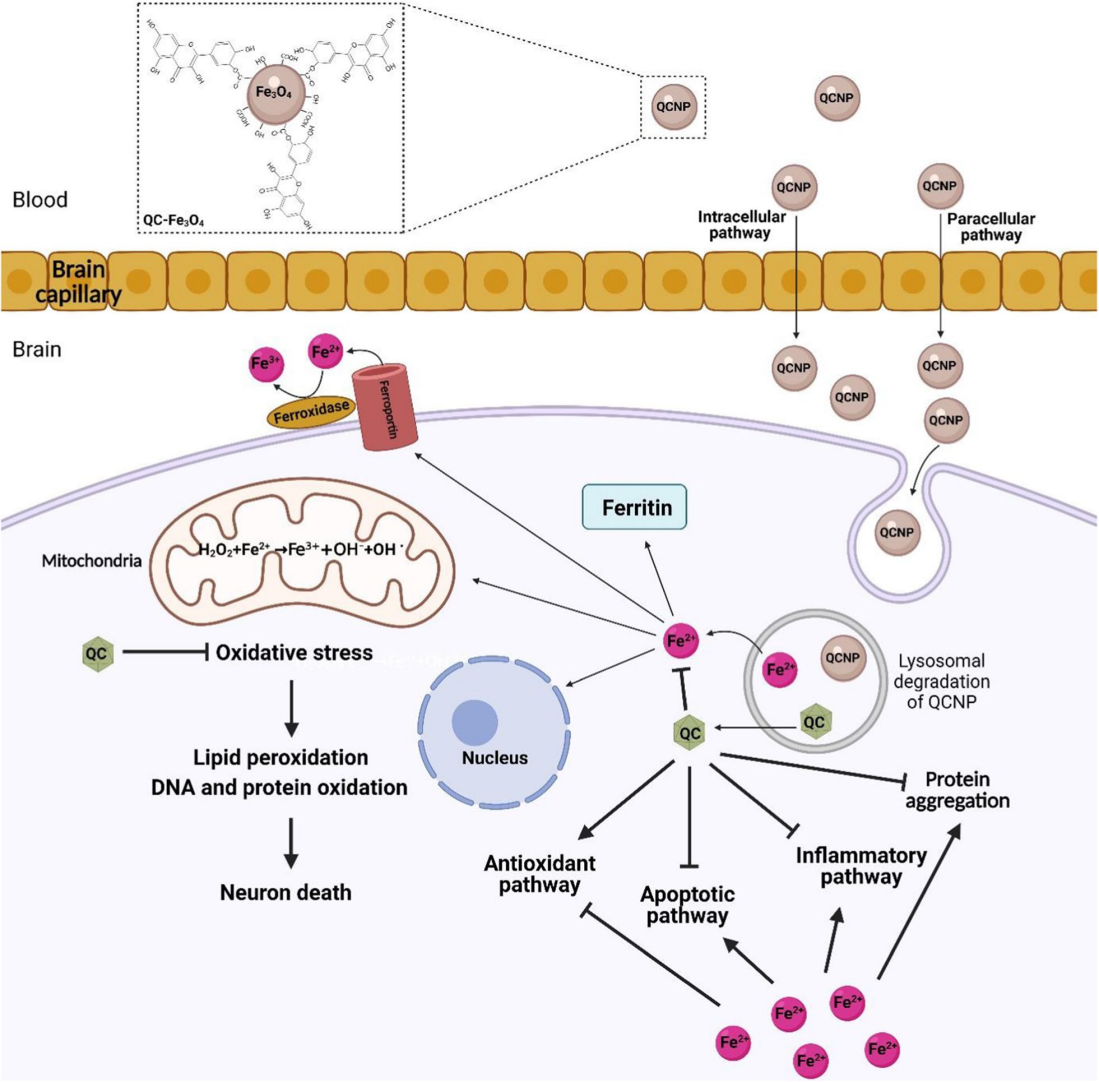
Cooperation of quercetin (QC) and dextran-coated IONPs in memory enhancement: QCIONPs cross BBB via paracellular or intracellular pathways. After the internalization of QCIONPs to neural cells, they are trapped in the lysosome. In the acidic pH of the lysosome, QCIONPs are decomposed. Excess iron raised from IONPs metabolism can be trapped by ferritin, transferred out of the cell through ferroportin, penetrate mitochondria and the nucleus. They can induce inflammation, protein aggregation, oxidative damage, and apoptosis. The released QC from QCIONPs suppresses the causes of neuronal death caused by neurodegenerative diseases and iron ions released from IONPs core. Also, QC prevents iron overload raised from IONPs degradation via iron chelation and radical scavenging. QC, quercetin; QCIONPs, QC conjugated with iron oxide nanoparticles; BBB, blood–brain barrier. This Figure was created by BioRender (https://biorender.com/)

At the same time, the released QC from QCIONP inhibits inflammation, apoptosis [248], protein aggregation [222, 227], and regulates antioxidant pathways to restore oxidative balance [181, 240]. Besides, QC via iron chelation and radical scavenging prevents iron overload raised from the metabolism of its carrier, Fenton reaction, and inhibits neuronal death (Fig. 7). QC is helpful to reduce toxicities caused by oral and IV administration of IONPs. It has been reported that QC could inhibit ferrous sulfate hepatorenal toxicity and decrease liver and renal tissue injury degree in rats [262]. In a study, the effects of QC on various mouse tissue injuries exposed to iron overload were studied. A diet containing QC revealed a significant reduction in liver and kidney iron content and a significant effect in suppressing iron overload-induced injury after administration of 500 mg/kg iron dextran for 45 days in a mouse model [263]. Another study compared the effect of deferoxamine as a conventional chelating agent and QC on iron overload in intestinal tissue of rats. This study showed QC improves small intestinal oxidative stress, iron-induced intestinal inflammation, apoptosis, and histopathological alterations, similar to deferoxamine [264]. In addition, protective effects of QC on intestinal damage due to ionizing radiation were studied in the rat model of radiation-induced ileitis and colitis. This study confirmed that QC significantly decreased oxidative stress and inflammatory damage in both ileum and colon tissues [265]. To the best of our knowledge, there are no studies show the effects of QC on anaphylactic shock induced by IV iron administration. Since the anti-allergic effects of QC on allergic diseases have been well established [266], it is suggested that studies should be designed on the protective effects of QC against iron anaphylaxis.

## Other iron-chelating compounds

There are several natural and synthetic compounds with iron-chelating activity and antioxidant properties that are used to overcome iron overload. Natural chelators contain a wide range of flavonoids such as QC [176] and microbial siderophores such as deferoxamine (DFO). In recent years, synthetic chelators such as deferasirox (DFX) and deferiprone (DFP) have been designed to mimic the effects of conventional chelators for clinical use [176]. Iron can bind to ligands possessing =O, –OH, –N, and –SH, which have the electron-donating property to form a coordinate bond [267]. Previous studies reported positive effects of the DFO on dementia-associated diseases such as AD, PD, and stroke [268]. DFO is an efficient drug for improving dementia-associated diseases [150]. Over the past 5 decades, DFO has been commonly used in patients suffering from iron overload [184]. DFO is a hexadentate iron chelator, contains hydroxamate functional groups. Therefore, each atom of iron can be surrounded by one molecule of DFO (1:1 ratio) to form a feroxamine complex. This natural iron chelator is produced by the Streptomyces species [267, 269, 270]. DFO has poor oral absorption and a very short biological half-life (20–30 min). Therefore, it should be injected subcutaneously or intravenously in doses less than 60 mg/kg/day for 8–12 h a day and at least 5 days a week. There is a probability of poor compliance in some patients that use this chelator. After absorbing, DFO chelates plasma iron and is excreted by urine and feces. DFO induces ferritin degradation by autophagy and subsequently eliminates excess iron in the cell [184, 270–272]. Due to high molecular weights (500–900 g/mol) and low lipid solubility, DFO cannot effectively pass across BBB [184, 273]. Although, Ward et al. reported that i.p administration of DFO (30 mg/kg) in ferrocene-loaded rats decreased iron content in the brain that represents DFO ability for crossing BBB [185]. DFO delivery to the brain through the intranasal route can relatively overcome the limitation of the BBB. Intranasal administration of DFO to the brain decreases pathological hallmarks of AD including Aβ, GSK-3β activity, and oxidative stress in the APP/PS1 mouse model of AD [274]. DFO treatment improves post-stroke cognitive dysfunction and long-term sensorimotor via a decrease in markers of oxidative stress, ferroptosis, BBB permeability, microglial activation in the rat model [275]. Despite DFO is an efficient natural chelator, it has a wide range of side effects including renal complications, dysfunction of auditory and visual systems, growth retardation, especially in children [276, 277], allergic reactions at the infusion site [278], and neurological side effects at high doses [271]. Some of these side effects are removed by lowering the dose [276, 277].

DFX is the first oral chelator, a tridentate iron chelator with a molecular weight of 373 g/mol and a half-life of 8–16 h. Each iron ion can be surrounded by two molecules of DFX (2:1 ratio) to form Fe-[DFX]_2_ complex [267, 271, 279]. DFX is designed in the form of dispersible tablets that are recommended once-daily dosing [271]. DFX has more absorption than DFO so its oral bioavailability is estimated at 70% on average. DFX is mainly excreted via bile and feces [279]. DFX has relatively low BBB penetration, and its affinity for iron is relatively weak. Thus the ability of DFX for chelation of brain iron likely is very low [92]. DFX treatment (25 mg/kg) improves dyshomeostasis of iron and Aβ and markedly prevents overexpression of TfR and ferritin. Moreover, DFX inhibits NF-κB activity induced by iron accumulation and oxidative stress in the aged rat brain and resulting in reducing inflammatory cytokines. These data suggest DFX can be used to ameliorate AD symptoms [280]. Systemic administration of DFX (20 mg/kg) induces neuroprotection in the 6-OHDA model of PD [281]. DFX decreases iron accumulation and oxidation products induced by intracranial hemorrhage in hemorrhagic stroke models. It can prevent apoptosis and autophagy by reducing the levels of apoptotic markers such as caspase-3, PARP, and autophagic markers such as LC3 and p62 [282]. However, high doses of DFX may lead to transient skin rash, abdominal pain, nausea [283], enhancing liver enzymes [271], and renal failure. Thus, duration treatment with DFX, renal and liver function are monitored [272].

DFP is very small with a molecular weight of 139 g/mol and bidentate iron chelator. Thus three molecules of DFP can attach to one iron atom (3:1 ratio) [267]. DFP is an orally active chelator, and the dose range is between 75 and 100 mg/kg/day that can be used three times daily in the forms of tablets or oral solution [271]. The half-life of DFP is 2–3 h and can be excreted by the urine. DFP is more affordable than DFX [270, 284]. Oral administration of DFP in early-onset PD patients decreased iron levels in the dentate and caudate nucleus and relatively improved motor symptoms. However, there was no improvement in cognitive symptoms [285]. DFP treatment (10 and 50 mg/kg/day) decreased BACE-1 expression, Aβ level, and tau phosphorylation without any effect on brain iron content and ROS. Although DFP reduces plasma iron and cholesterol levels [286]. It can be suggested that the application of simultaneous antioxidant agents with DFP is required to reinforce the iron chelation of DFP. In some cases, DFP has reversible side effects such as gastrointestinal symptoms, musculoskeletal pain [284], enhancing liver enzymes [271], and neutropenia or agranulocytosis thus regular monitoring of white blood cells and liver function is required [272]. Table 2 presents a comparison between different types of iron chelators^16^.

**Table 2:** Comparison between QC and conventional iron chelators

Iron chelating agents	Quercetin (QC)	Deferoxamine (DFO)	Deferasirox (DFX)	Deferiprone (DFP)
Type (source)	Natural (flavonoid) [176]	Natural (siderophore) [176]	Synthetic [176]	Synthetic [176]
Molecular weight	302.236 g/mol (see Foot note link 13)	500–900 g/mol [273]	373 g/mol [267]	139 g/mol [267]
Half-life	11–28 h [176]	20–30 min [270]	8–16 h [270]	2–3 h [270]
Routes of excretion	Urinary [172], fecal [171]	Urinary, fecal [270] [299]	Fecal [279]	Urinary [270]
Structure				
Recommended dose	500–1000 mg/day for short time (web ref, 14) 500 mg twice daily for 12 weeks (see Foot note link 15)	20–60 mg/kg/day over 8–24 h [271]	20–40 mg/kg/day once daily [271]	75–100 mg/kg/day in three divided doses [271]
Administration	Oral and intravenously (see Foot note link 15). powder and capsule (see Foot note link 14)	Subcutaneous and intravenous [267]	Oral [279](dispersible tablet) [271]	Oral [92]tablets and solution [271]
Stoichiometry (chelator:iron)	1:1 2:1 3:1 [18, 189]	1:1 [292]	2:1 [292]	3:1 [292]
Adverse effects	Generally safe. at doses, more than 1000 mg/day may cause headaches, stomach aches, and tingling sensations (see Foot note link 14)	Neurological side effects at high doses [271], ocular and auditory toxicity, renal complications, growth retardation [276, 277], local allergic reactions [278]	Skin rash, gastrointestinal complications, [283], enhancing liver enzymes [271]	Gastrointestinal complications, musculoskeletal pain, neutropenia [284], and enhancing liver enzymes [271]
Advantages	No side effects in desirable doses and duration of medication (web ref, 14 and 15), supplied by natural sources, and longer plasma half-life than current chelators (e.g. DFO, DFX, and DFP)	Long-term experience and data available [299]	Orally active, once-daily dosing [299] and long-plasma half-life [270]	Orally active [92], low molecular weight, and high ability to penetrate tissues
Disadvantages	Requires high dose and low BBB crossing	Not absorption from the gastrointestinal tract [270], rapidly clearance and requires to prolong infusion [300], and poor compliance [184]	Expensive [299] and requires monitoring renal and liver function [272]	Moderate-plasma half-life [270], requires three times daily dosing and probability of negative effects [271], limited experience and data (see foot not link 16), and requires assessment of complete blood counts [270]
FDA approval	No	Yes [278]	Yes [278]	Yes, except the United States and Canada [270]

QC seems to be more desirable than other chelators because of no side effects and a longer half-life. Besides, the availability of QC as a natural compound, which is widely present in meals, is an important advantage. In addition, QC can ameliorate the toxicity effects of other nanoparticles such as zinc oxide nanoparticles (ZnO-NP). In this regard, QC showed protective effects on ZnO-NP-induced mouse Sertoli cell (TM4 cell line) through anti-apoptotic, antioxidant, and autophagic induction properties [287]. An in vivo study showed the protective role of early treatment with QC and l-arginine against cardiotoxicity induced by ZnO-NP in rats [288]. Another study showed modulation effects of QC as a free-radical quencher against toxicity of copper oxide nanoparticles in rat liver [289]. However, QC is not an FDA-approved supplement to attenuate metal toxicity and ameliorate related diseases. Thus, more studies are required to prove its effectiveness on iron overload and the resulting consequences.

## Conclusion and prospect

Iron is an essential mineral for general health and its deficiency is the most common form of nutrient deficiency [290]. There is a strong link between iron deficiency and cognitive functions [291]. In general, the role of iron in the metabolism of most tissues, especially the brain, is like a double-edged sword. Iron is needed as a redox-active metal to produce ATP-producing enzymes in the brain cells, but excessive iron causes oxidative stress, protein accumulation, and ferroptosis [101]. There is a significant link between the accumulation of brain iron and diseases such as AD, PD, and stroke. The amount of iron in certain parts of the brain can be used as an indicator to track the degeneration of the CNS. However, it is not clear whether iron accumulation in the brain of patients is a primary phenomenon in the initiation of neurodegenerative diseases or whether iron accumulation is a secondary event [292].

In recent years, a lot of information has been obtained about the toxicity of IONPs through various studies. But it is not yet completely certain that whether this information also applies to complex biological fluids. On the other hand, due to laboratory errors and different laboratory conditions of various studies, comparing the results of these studies will be somewhat unreliable. It is clear that to use IONPs with unique physical properties, a very high but perfectly acceptable barrier provided by regulatory bodies must be overcome [1]. The number of publications related to IONPs has increased strongly over the years, and their toxicity in medical applications has become a matter. Despite a huge and increasing number of publications about the application of IONPs in biomedicine, there is a significant gap in knowledge on the toxicity profile of these promising particles and their suggestion for safe use in many aspects of medical engineering in the future. One strategy for long-term usage of IONPs with minimum toxicity is the delivery of IONPs accompanied by iron chelators. Simultaneous application of iron chelators can inhibit neurotoxicity induced by IONPs metabolism. In this review, we suggested the simultaneous use of QC in combination and especially conjugated form can be a useful strategy to reduce brain oxidative damages and aggregation of IONPs. On the other hand, QC requires a delivery system to show its maximum efficiency. Based on previous studies, we have proposed IONPs as a nanocarrier that enhances the bioavailability of QC in the brain.

In addition, despite QC is a strong antioxidant and iron chelator, it shows prooxidant properties in some of the studies [293–295]. QC is oxidized into o-quinone/quinonmethide (QQ) during the protection against oxidative stress via free radical scavenging. QQ has four tautomeric forms including ortho-quinone and three quinone methides. QQ has high reactivity toward thiol groups, which leads to arylation of protein thiols and impairment in several vital enzymes. Oxidized QC can be recycled in an interplay with other antioxidants such as ascorbate and NADH, called antioxidant networking. Ascorbate and NADH recycle QQ to the parent phenolic acid and it becomes available again for the antioxidant network. However, during this reaction, ascorbate and NADH becomes oxidized giving dehydroascorbate (DHA) and NAD^+^, respectively. QQ is toxic in the absence of GSH because GSH can produce reversible GSQ adducts including 6-GSQ and 8-GSQ [293, 296]. Therefore, administration of ascorbate supplementation is suggested during QC ingestion at a high dose level. Co-administration of ascorbate and QC exerts a synergistic action. In the pathological condition, there is an imbalance between oxidants and antioxidants levels that is accompanied by a decrease in GSH levels. Under this condition, low levels of GSH contribute to the arylation of other thiol proteins by QQ and cell damage [293, 297]. However, various studies indicated optimal concentrations of QC increase GSH levels [222, 224, 238]. Besides, in vitro studies reported short-term treatment with QC exerts antioxidant effects via a decrease in H_2_O_2_, whereas extending treatment duration represents prooxidant activity of QC via an increase in O_2_^−^, which was accompanied by a decrease in GSH levels [294, 295]. Thus, whether QC acts as an antioxidant or as a prooxidant depends on the dose and time of QC exposure [294]. In addition, QC like other dietary factors mainly affects iron absorption, but the role of flavonoids on iron homeostasis is complicated. QC as one of the main flavonoids inhibits iron absorption in the duodenum. It is believed that its power to chelate iron is mainly responsible for inhibiting iron absorption. In contrast, it has been reported QC can act as a substrate for Dcytb and providing more Fe^2+^ for cellular uptake by DMT1 [21]. Therefore, a safe and effective nano-based delivery system has been needed to improve QC limitations and decrease the dose of this compound in clinical application [177].

In conclusion, since the toxicity of IONPs has become a major challenge in medical applications, it is essential to provide a solution to minimize this toxicity. Simultaneous use of QC as a natural iron chelator in combination and especially conjugated form can be an effective strategy to reduce toxicity and aggregation of IONPs in clinical application. This not only helps to reduce the toxicity of IONPs but also increases the bioavailability of QC. This is a double benefit. Despite the limitations of the application of QCSPIONs in animal models, we hope that the present review opens a new window for using this compound in clinical trials on a large scale.

## Data Availability

Not applicable.

## Abbreviations

Ach: Acetylcholine
AD: Alzheimer’s disease
Apo-Tf: Apo-transferrin
APP: Amyloid precursor protein
BBB: Blood–brain barrier
BCB: Blood-CSF barrier
BMI: Body mass index
CDK: Cyclin-dependent kinase
CME: Clathrin-mediated endocytosis
CNS: Central nervous system
COMT: Catechol-O-methyl transferase
CSF: Cerebrospinal fluid
Dcytb: Duodenal cytochrome B
DFO: Deferoxamine
DFP: Deferiprone
DFT: Density functional theory
DFX: Deferasirox
DMT1: Divalent metal transporter 1
GPX: Glutathione peroxidase
HCP1: Heme carrier protein 1
HO-1: Heme oxygenase-1
HNE: Hydroxynonenal
IONPs: Iron oxide nanoparticles
IRE: Iron-responsive element
IRP: Iron-regulatory proteins
IV: Intravenous
LIP: Labile iron pool
LTP: Long-term potentiation
MAO: Monoamine oxidase
MDA: Malondialdehyde
MRI: Magnetic resonance imaging
MTC: Magnetic targeted carriers
NEIL: Nei like DNA glycosylase
NFTs: Neurofibrillary tangles
NP: Nanoparticle
NTBI: Non-transferrin bound iron
PD: Parkinson’s disease
PEG: Polyethylene glycol
PEO: Polyethylenoxide
PVA: Polyvinyl alcohol
QC: Quercetin
QCSPIONs: Quercetin conjugated superparamagnetic iron oxide nanoparticles
QSM: Quantitative susceptibility mapping
RNS: Reactive nitrogen species
ROS: Reactive oxygen species
SOD: Superoxide dismutase
SPIONs: Superparamagnetic iron oxide nanoparticles
TAC: Total antioxidant capacity
USPIONs: Ultra-small superparamagnetic iron-oxide nanoparticles
UTR: Untranslated region

## Footnotes

1 https://www.drugs.com/history/combidex.html

2 https://www.ncbi.nlm.nih.gov/books/NBK23402

3 https://www.accessdata.fda.gov/drugsatfda_docs/label/2009/017441s171lbl.pdf

4 https://reference.medscape.com/drug/infed-iron-dextran-complex-342173

5 https://www.drugs.com/pregnancy/iron-dextran.html

6 https://pubchem.ncbi.nlm.nih.gov/compound/Iron-dextran#section=Toxicity-Summary

7 https://www.accessdata.fda.gov/drugsatfda_docs/label/2017/021135s032lbl.pdf

8 https://www.medicines.org.uk/emc/product/5911/smpc#gref

9 https://www.rxlist.com/venofer-drug.htm#indications

10 https://www.drugs.com/pro/feridex.html

11 https://www.accessdata.fda.gov/drugsatfda_docs/label/2009/022180lbl.pdf

12 https://go.drugbank.com/drugs/DB06215

13 https://pubchem.ncbi.nlm.nih.gov/compound/Quercetin

14 https://www.healthline.com/nutrition/quercetin

15 https://www.webmd.com/vitamins/ai/ingredientmono-294/quercetin

16 https://slideplayer.com/slide/8024705
